# Lactate and lactylation: mechanisms, function, diseases, and therapeutic targets

**DOI:** 10.1186/s43556-026-00472-x

**Published:** 2026-05-20

**Authors:** Xiuping Wang, Cheng Liu, Yue Chen, Yuqing Fang, Jingyi Li, Yu Liu, Yu Pan, Suya Bao, Xiwen Wang, Yi Liu, Hengwei Liu

**Affiliations:** 1 Department of Obstetrics and Gynecology, Union Hospital, Tongji Medical College, Huazhong University of Science and Technology, Wuhan, 430022 China; 2 Department of Obstetrics and Gynecology, Zhongnan Hospital of Wuhan University, Wuhan, 430071 China; 3 Department of Gynecology and Obstetrics, Renmin Hospital of Wuhan University, Wuhan, Hubei Province China

**Keywords:** Lactate metabolism, Lactylation, Post-translational modifications, Metabolic reprogramming, Epigenetic regulation

## Abstract

The paradigm shift of lactate from a mere metabolic byproduct to a pleiotropic signaling molecule, coupled with the discovery of lactylation, provides a crucial framework for understanding how metabolic reprogramming drives systemic pathology. In this review, we systematically delineate the dynamic equilibrium of lactate homeostasis and the evolution of the lactate shuttle theory, exploring its multidimensional roles as a metabolic substrate, signal transducer, and immunomodulator. Furthermore, we summarize how lactylation orchestrates diverse pathophysiological processes across major organ systems, including metabolic dysfunction and fibrosis in the cardiovascular system, neuroinflammation and apoptosis in the central nervous system, and microenvironment-driven injury across the respiratory, digestive, and urinary tracts. Notably, we highlight the female reproductive system as a unique physiological model for investigating metabolic-epigenetic crosstalk, detailing how histone and non-histone lactylation contribute to the progression of various gynecological pathologies and critical reproductive processes. Finally, we evaluate the clinical translational potential of lactate-related biomarkers and lactylation-targeted therapeutics. Ultimately, this comprehensive framework underscores lactate and its mediated modifications as fundamental epigenetic regulators of cellular function and promising pharmacological targets for precision medicine.

## Introduction

Metabolic reprogramming refers to the cellular adaptation to changes in the microenvironment by adjusting the redistribution of glucose, lipids and amino acids to meet increased energy and biosynthetic demands [1]. In inflammatory-related diseases, the proliferation of inflammatory cells forms a malignant inflammatory microenvironment, prompting cells to undergo metabolic reprogramming, altering the energy supply mode of cells within the microenvironment, thus influencing the progression of the disease [2]. Even under aerobic conditions, abnormally proliferating cells preferentially convert glucose into pyruvate to produce lactate, accompanied by limited adenosine triphosphate (ATP) generation, a process known as aerobic glycolysis. This low-energy metabolic pathway was first observed by Otto Warburg and is therefore also referred to as the "Warburg effect" [3]. The Warburg effect is prominently associated with diverse pathophysiological processes involving oncogenesis and inflammation [4–7].

The accumulation of lactate, regulated by numerous key enzymes, is the endpoint of the Warburg effect. Lactate was previously regarded as a major fatigue agent or metabolic toxin primarily derived from glycolysis, acutely accumulating during exercise and chronically in tumor microenvironments (TME) and inflammatory sites. Since the lactate revolution of the 1970 s, lactate has been repositioned as a distinctive energy source and crucial signaling molecule, recognized as a major metabolic intermediate. Recent studies have found that it can mediate immune-inflammatory responses, angiogenesis, fibrosis, and other processes [4]. As a pro-angiogenic factor, lactate promotes angiogenesis by stabilizing hypoxia-inducible factor 1-alpha (HIF-1α) and increasing vascular endothelial growth factor (VEGF) expression [5]. Moreover, lactate has the ability to both directly and indirectly suppress anti-tumor responses. Research has shown that lactate-induced HIF-1α can drive the differentiation of myeloid-derived suppressor cells (MDSCs) into tumor-associated macrophages (TAMs) by modulating the expression of inducible nitric oxide synthase (iNOS) and arginase-1 (ARG1), thereby promoting the suppression of adaptive immune responses [6, 7]. Three major milestones have shaped lactate metabolism research: the discovery of the "Warburg effect" by Otto Warburg in 1921, George Brooks' proposal of the "lactate shuttle theory" in 1984, and the 2019 discovery by Yingming Zhao's team of lactylation, a novel post-translational modification (PTM) that modifies histones and functional proteins, providing new insights into gene regulation and expression [8–10].

Building upon these milestones, it is now evident that lactate acts as a vital intermediary, translating metabolic signals into stable cellular programs via lysine lactylation. This review systematically delineates how lactylation bridges metabolic reprogramming and multisystemic pathogenesis, driving diverse conditions such as tissue fibrosis, neuroinflammation, and autoimmune dysfunction across various organ systems. Notably, we highlight the female reproductive tract as a core physiological model, given its naturally acidic niche and high-intensity metabolic flux. By integrating metabolic mechanisms and immune crosstalk, we detail how lactylation orchestrates the progression of major gynecological pathologies and critical reproductive processes. Ultimately, this framework underscores the significant regulatory roles of lactate and lactylation in cellular biology, positioning them as promising pharmacological targets for precision medicine.

## Lactate homeostasis

### The origin and fate of lactate

**Fig. 1:**
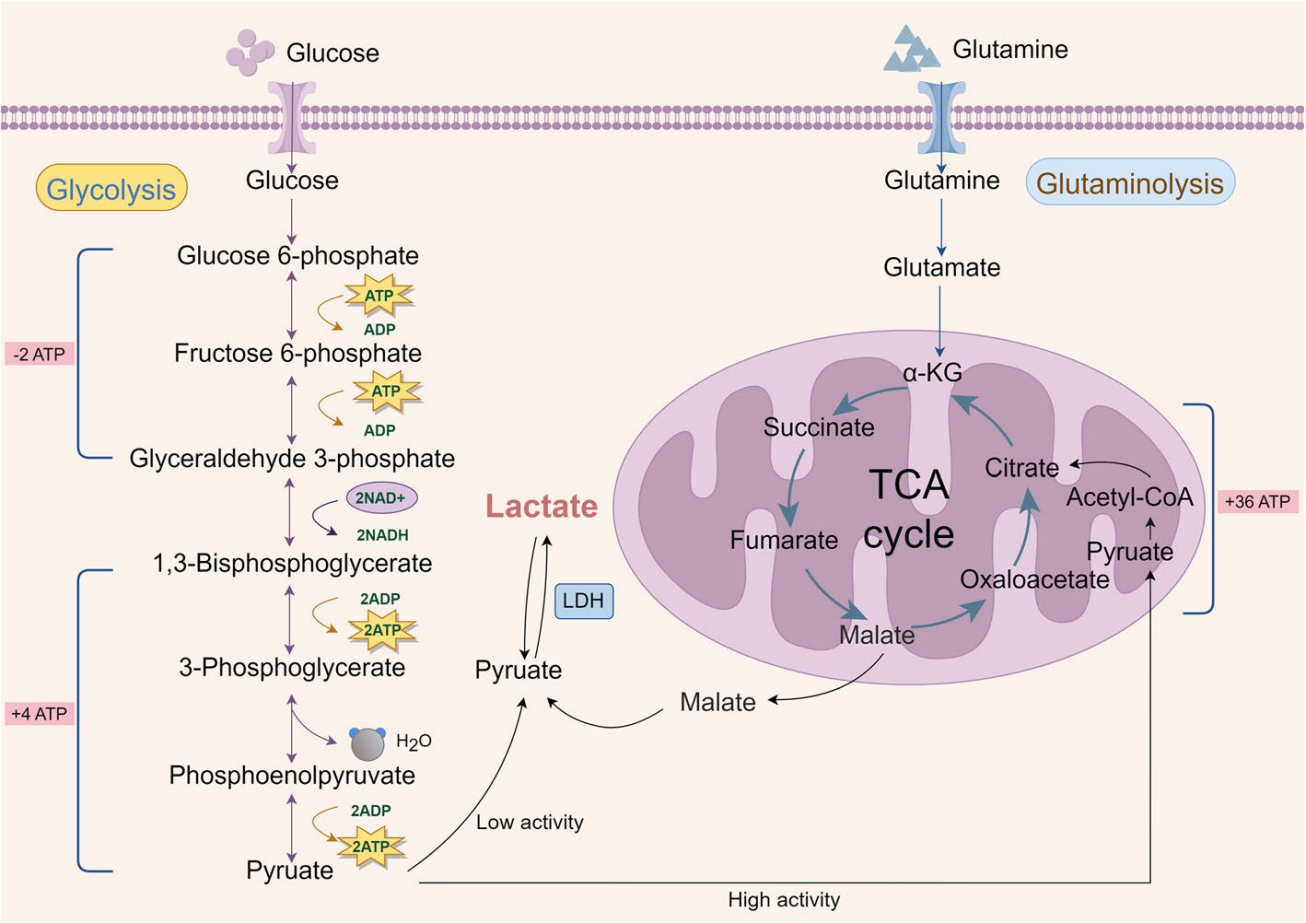
Lactate metabolism in cells**.** Lactate production primarily occurs through glycolytic and glutaminolytic pathways. During lactate clearance, lactate is first oxidized to pyruvate, which then enters the mitochondria to be metabolized through the TCA cycle. This process leads to the irreversible elimination of lactate

Lactate production occurs primarily through the glycolytic pathway. Under anaerobic conditions, the inhibition of the tricarboxylic acid (TCA) cycle activates glycolysis to compensate for reduced ATP production. During glycolysis, glucose is converted into two molecules of pyruvate, generating two ATP molecules and two nicotinamide adenine dinucleotide (NADH) molecules. Through the fermentation process, pyruvate and NADH are reduced to lactate. Consequently, each glucose molecule yields two ATP molecules and two lactate molecules without oxygen consumption [11]. However, in tumor cells with high expression of GLUT1, increased glucose uptake occurs regardless of oxygen availability. These cells primarily use lactate dehydrogenase (LDH) to catalyze the conversion of pyruvate to lactate, meeting 40–75% of their energy needs through this aerobic glycolysis pathway. The remaining energy is generated through mitochondrial respiration and oxidative phosphorylation (OXPHOS) pathways [12]. Although glycolysis generates only 2 ATP molecules compared to 36 ATP molecules from OXPHOS, tumor cells take up approximately 10 times more glucose than normal cells, resulting in a 10–13% increase in total ATP production compared to normal cells. This surplus ATP, produced via glycolysis, supplies tumor cells with essential metabolic intermediates (e.g., glucose-6-phosphate, glyceraldehyde-3-phosphate, and 3-phosphoglycerate) to meet the heightened biosynthetic demands required for proliferation [13]. In addition to glycolysis, glutaminolysis represents another significant source of lactate production in tumor cells. Tumor cells express high levels of glutaminase 1 (GLS1), an enzyme regulated by the oncogenic transcription factor c-Myc, which converts glutamine into glutamate through a deamination reaction in the mitochondria. This glutamine uptake occurs via amino acid transporters such as the amino acid transporter type 2 (ASCT2) and sodium-coupled neutral amino acid transporter 5 (SN2) [11, 14]. Glutamate is subsequently converted into α-ketoglutarate (α-KG), a key intermediate in the TCA cycle, through the actions of either glutamate dehydrogenase (GDH) or transaminases (TAs), which utilize alanine or aspartate as substrates. α-KG is a crucial metabolite that supports ATP production and replenishes substrate in the TCA cycle [15, 16]. With the cycle, carbon derived from glutamine is converted into oxaloacetate, which can be used to generate citrate and acetyl-CoA. Acetyl-CoA, in turn, acts as a precursor for lactate [14](Fig. 1). In SF188 glioblastoma cells, approximately 90% of oxaloacetate is synthesized from glutamine. Moreover, the metabolic conversion of glutamine to lactate produces a substantial amount of NADPH, which is critical for the biosynthesis of fatty acids [17].

Lactate clearance primarily occurs via two distinct pathways. In one of these pathways, lactate is converted back into pyruvate, which then enters the mitochondria and undergoes metabolism through the TCA cycle, facilitated by the enzyme pyruvate dehydrogenase (PDH). This process results in the irreversible removal of lactate. The second pathway involves the absorption of lactate by the liver, where it is converted into glucose via gluconeogenesis. This glucose then re-enters circulation to provide energy for tissues in need, a process known as the Cori cycle [18].

### The lactate shuttle theory

**Fig. 2:**
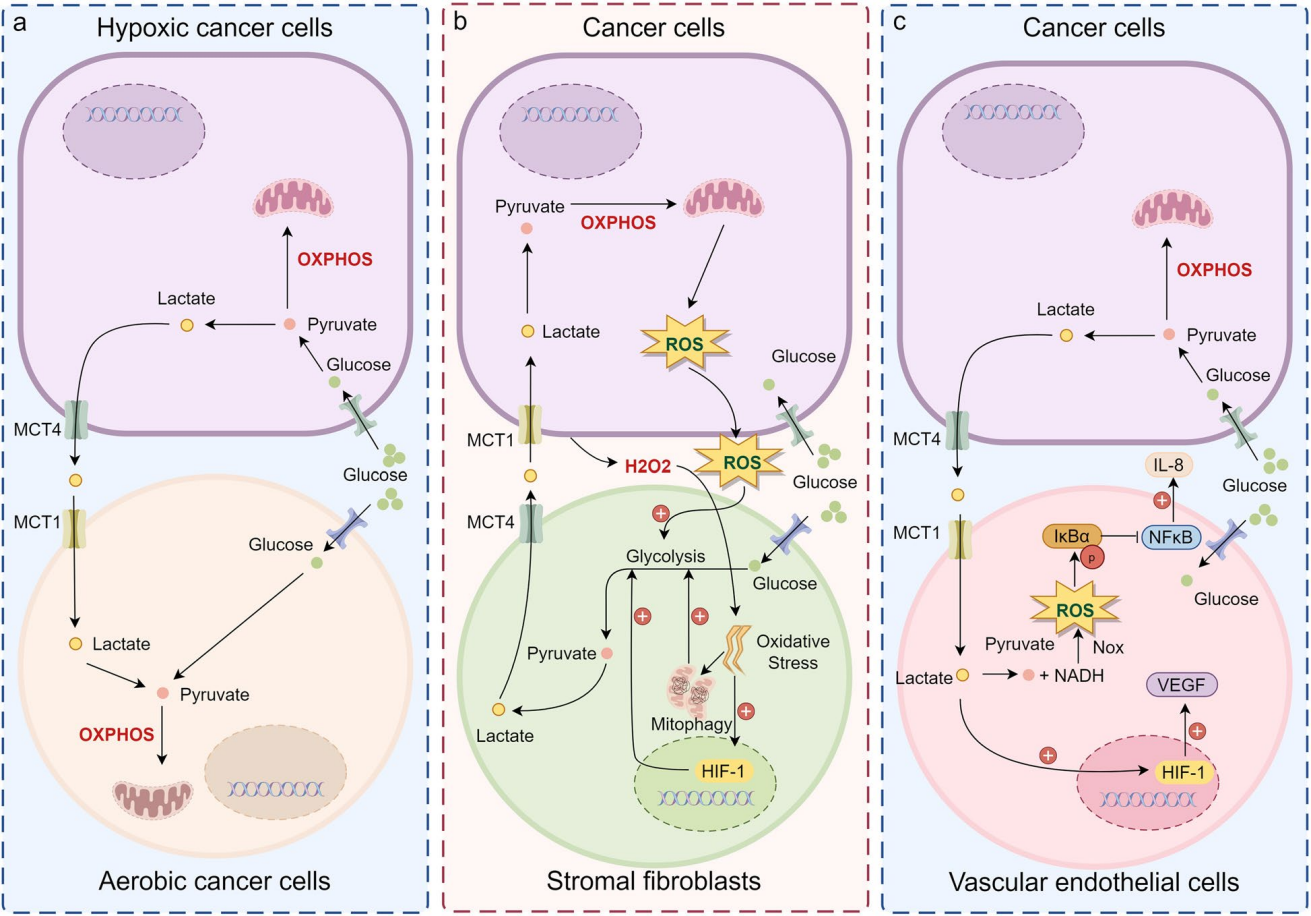
Lactate shuttle mediated by MCTs. **a**. Lactate shuttles between aerobic and hypoxic cancer cells. Hypoxic cancer cells convert ingested glucose into pyruvate via glycolysis and subsequently convert pyruvate into lactate through LDHA, which is then exported via MCT4. Aerobic cancer cells absorb lactate through MCT1 and convert it back to pyruvate via intracellular LDHB, integrating it into oxidative phosphorylation for energy metabolism. **b**. Lactate shuttles between cancer cells and fibroblasts. Cancer cells secrete hydrogen peroxide, creating a pseudo-hypoxic environment that induces oxidative stress, leading to mitophagy and HIF-1 overexpression in fibroblasts, resulting in increased lactate production and export via MCT4. Cancer cells absorb lactate through MCT1 and convert it to pyruvate, entering the oxidative phosphorylation pathway for utilization. **c**. Lactate is exchanged between cancer cells and vascular endothelial cells. Cancer cells export lactate via MCT4, while endothelial cells import lactate through MCT1. This lactate uptake induces the phosphorylation of IκBα, which activates the NF-κB/IL-8 signaling pathway, thereby promoting angiogenesis in cancer cells

The lactate shuttle theory posits that lactate functions as a metabolic intermediate shuttled between glycolytic (producer) and oxidative (consumer) cells, conferring diverse biological roles [4]. This concept involves both intercellular and intracellular exchange, with lactate functioning as an energy substrate for highly oxidative tissues such as the heart, while also acting as a gluconeogenic precursor in the liver and kidneys [19]. Brooks' expansion of the lactate shuttle theory in 1998 introduced the notion of intracellular shuttling, including exchanges between cytosol, mitochondria, and peroxisomes, highlighting the interconnectedness of glycolysis and aerobic metabolism [20]. Most intercellular and intracellular lactate shuttles are driven by concentration or pH gradients or redox states, mainly mediated by transport proteins (such as MCT1, MCT4) and receptors (such as GPR81). MCTs regulate the transfer of lactate between hypoxic and aerobic cancer cells within tumors, as well as between tumor cells and the surrounding stromal and endothelial cells. Given the presence of both aerobic and hypoxic regions in tumors, cancer cells are categorized into aerobic and hypoxic subpopulations [21]. Hypoxic cancer cells release lactate, a byproduct of glycolysis, through MCT4, while aerobic cancer cells take up lactate via MCT1. Inside the aerobic cells, lactate is converted back to pyruvate by LDHB. This pyruvate is subsequently directed into the oxidative phosphorylation pathway, where it contributes to cellular energy production [22]. Lisanti et al.'s discovery of the reverse Warburg effect in 2009 further elucidated the role of lactate shuttling in tumor microenvironments. Cancer cells secrete hydrogen peroxide, creating a pseudo-hypoxic environment that induces oxidative stress. This stress triggers an increase in hypoxia-inducible factor 1 (HIF-1) expression in stromal fibroblasts, leading to elevated MCT4 expression and a metabolic shift towards glycolysis. Consequently, stromal fibroblasts produce and release lactate, which is then taken up by cancer cells for energy utilization [23–25]. In endothelial lactate shuttling, lactate released by cancer cells through MCT4 is taken up by endothelial cells via MCT1. This uptake of lactate initiates the phosphorylation of IκBα, which in turn activates the NF-κB/IL-8 signaling pathway, ultimately enhancing angiogenesis in tumor cells [26](Fig. 2).

Beyond cancer, lactate shuttling also plays significant roles in various physiological processes. In the female mouse reproductive system, ovarian follicular fluid exhibits higher lactate concentrations compared to the oviduct and uterine fluid [27]. In the ovary, excessive glycolysis in granulosa cells can lead to the accumulation of pyruvate and lactate, which can serve as substrates for oocyte oxidative phosphorylation [28, 29]. In the fallopian tube, MCT1 and MCT4 are specifically expressed on the cell membrane of ciliated cells in the ampulla and isthmus. Ciliated cells of the fallopian tube utilize lactate produced by adjacent non-ciliated cells as an energy source through MCT1. However, the direction of lactate transport mediated by MCT4 and its functional significance in ciliated cells of the fallopian tube remain to be fully elucidated [30]. In the vaginal cavity, lactobacilli metabolize glycogen from vaginal epithelial cells to produce lactate, creating an acidic environment that helps prevent vaginal infections [31]. Additionally, the vagina epithelium itself may produce lactate through glycolysis. The expressed MCT1 in the vaginal epithelium facilitates the transport of lactate to the vaginal lumen or subepithelial region, where it can be utilized as an energy source by sperm [30, 32]. Finally, in the uterine endometrium, lactate shuttling plays a role in decidualization. Decidualization is a process driven by glycolysis, and local lactate shuttling has been demonstrated in stromal cells. Studies have found that during decidualization, the Pi3k-Akt signaling pathway is activated in mouse uterine endometrium and influences MCT4 expression via HIF-1α signaling pathway. Furthermore, in vitro decidualization or lactate treatment processes, inhibition of MCT1 with CHC (a specific inhibitor targeting MCT1) reduces lactate-dependent proliferation of undifferentiated endometrial stromal cells and impedes their morphological transformation of stromal cells, suggesting the importance of lactate shuttling in this process [33].

### The evolution of lactate metabolism function

#### Lactate as a metabolic fuel

Recent research has redefined lactate, previously considered a metabolic waste product, as a valuable energy source. Lactate, as a significant substrate for energy metabolism, can be converted to pyruvate via the action of LDH in the process of glycolysis, thereby generating a certain amount of ATP. This pathway for energy production is crucial for cells and tissues under conditions of insufficient oxygen supply. Hui et al. demonstrated, using isotopically labeled 13C-lactate, that lactate is indeed a major fuel for the TCA cycle in both normal and tumor tissues [34]. Faubert et al. injected [U-13C] glucose and [3-13C] lactate into mice bearing HCC15 non-small-cell lung cancers (NSCLCs) xenografts. They found that both glucose and lactate contributed equally to pyruvate, but lactate made a more prominent contribution to the TCA cycle, with labeled TCA cycle intermediates citrate, glutamate, and malate being twice as high [35]. Furthermore, lactate decouples glycolysis and the TCA cycle, allowing these two pathways to operate independently. Most of the pyruvate generated from glycolysis is converted to lactate with the assistance of LDH and monocarboxylate transporters (MCTs) and released into the bloodstream. In the human body, the expression of glucose transporters (GLUTs), which facilitate the absorption of glucose into metabolism, is highly restricted, with the highest expression found in the brain and activated immune cells, and less in other tissues. However, MCTs are almost universally expressed, allowing lactate to be freely utilized as an energy source by all cells in the body. Additionally, glucose is retained to meet the higher energy demands of the organism [11]. When blood glucose is low or lactate levels are high, the brain can utilize lactate to support neural activity [36]. Lactate plays a crucial role during neuronal depolarization, with astrocytes storing most of the brain's glycogen. Upon neuronal activation, glycogen in astrocytes is converted to lactate, which is then transferred to neurons and converted to pyruvate, further facilitating energy production to meet the energy demands required for neuronal depolarization [37]. In the cumulus-oocyte complex (COC), lactate serves as a metabolic intermediary between the aerobic glycolysis occurring in granulosa cells and the oxidative phosphorylation in the oocyte. Lactate dehydrogenase A (LDHA) in granulosa cells converts a portion of the pyruvate produced during glycolysis into lactate, which is then transferred to the oocyte. Inside the oocyte, lactate dehydrogenase B (LDHB) reconverts lactate into pyruvate to fuel energy production. Furthermore, the high expression of MCTs on the oocyte membrane facilitates the uptake of energy precursors from the follicular fluid [28, 38].

#### Lactate mediates cell signaling transduction

Beyond its role as a metabolic byproduct, lactate has emerged as a multifunctional signaling molecule with broad implications. Lactate can regulate immune inflammatory responses, angiogenesis, and fibrosis [39]. By directly binding to the transmembrane domain of the mitochondrial antiviral signaling protein (MAVS), lactate inhibits the assembly of MAVS signalosome, thereby suppressing the sugar metabolism-mediated RIG-I-like receptor (RLR) signaling pathway and subsequent type I interferon production [40, 41]. Lactate acts as a novel regulator for c-Jun in myeloid cells, protecting it from degradation by the ubiquitin ligase F-box and WD repeat domain-containing 7 (FBW7). This protection facilitates the activation of c-Jun-driven Cox2 expression, which in turn promotes the differentiation of monocytes into granulocytic myeloid-derived suppressor cells (G-MDSCs) or M2-polarized tumor-associated macrophages (TAMs). Consequently, this process contributes to immune evasion in the tumor microenvironment [42]. Furthermore, lactate activates G-protein coupled receptor 81 (GPR81/HCAR1) to act as a signaling molecule. Lactate produced by cancer cells can act through both paracrine and autocrine pathways. It activates GPR81 not only on cancer cells but also on immune cells, endothelial cells, and adipocytes within the tumor stroma. This activation promotes angiogenesis, immune evasion, and resistance to chemotherapy, thereby facilitating tumor progression and metastasis [43]. Additionally, lactate can induce the expression of GPR81 in cancer cells by activating the transcriptional complex Snail/EZH2/STAT3 [44]. Lactate-mediated signaling through GPR81 can synergize with insulin to reduce intracellular cyclic AMP (cAMP) levels and lipid breakdown in adipocytes [45]. Lactate inhibits Toll-like receptor 4 (TLR4)-induced activation of NF-κB and inflammasome in macrophages, monocytes, and other cell types, as well as downstream transcription of pro-IL-1β and pro-IL-18. [46, 47].

####  Lactate modulates immune cell function

During inflammation, immune cells not only generate significant amounts of lactate via glycolysis but also detect lactate through receptors such as GPR81 or MCTs on their cell surface. This lactate sensing modulates their cellular metabolism, ultimately influencing their phenotype and driving them towards either pro-inflammatory or anti-inflammatory states [39, 48]. In macrophages stimulated with lipopolysaccharide (LPS), lactate administration leads to a metabolic shift, characterized by a decrease in the extracellular acidification rate (ECAR) and an increase in the oxygen consumption rate (OCR). This metabolic reprogramming facilitates a shift from a pro-inflammatory to an anti-inflammatory phenotype, as demonstrated by decreased inflammasome assembly, reduced LPS-induced cytokine secretion, and impaired migration of monocytes and macrophages [49]. Furthermore, lactate can decrease the activity of phosphofructokinase (PFK), favoring the dissociation of its tetrameric form into less active dimers. This metabolic change results in decreased glycolytic flux and a reduction in the pro-inflammatory phenotype of monocytes [50]. However, the effects of lactate on immune cells can be multifaceted. In macrophages derived from human monocytes, lactate has been shown to stimulate the expression of MD-2 (a Toll-like receptor 4 co-receptor), NF-κB signaling transduction, and downstream inflammation gene transcription, exhibiting pro-inflammatory effects[51]. Additionally, lactate induces the mobilization of neutrophils from the bone marrow by increasing the release of CXCL1, CXCL2, along with granulocyte colony-stimulating factor (G-CSF) [52]. Lactate also influences T cell behavior by impairing their migration, which results in their accumulation at sites of inflammation. Additionally, lactate stimulates the secretion of IL-17 by CD4^+^ T cells while simultaneously inhibiting the cytotoxic activity of CD8^+^ T cells [53]. Lactate enhances fatty acid synthesis and promotes the production of IL-23/IL-17 by CD4^+^ T cells, serving as a pro-inflammatory signal and sustaining the chronic inflammatory process [54]. When activated CD4^+^ T cells are treated with lactate, there is an increase in nuclear translocation of PKM2 and enhanced phosphorylation of STAT3, leading to the polarization of CD4^+^ T cells towards the Th17 phenotype [55].

**Fig. 3:**
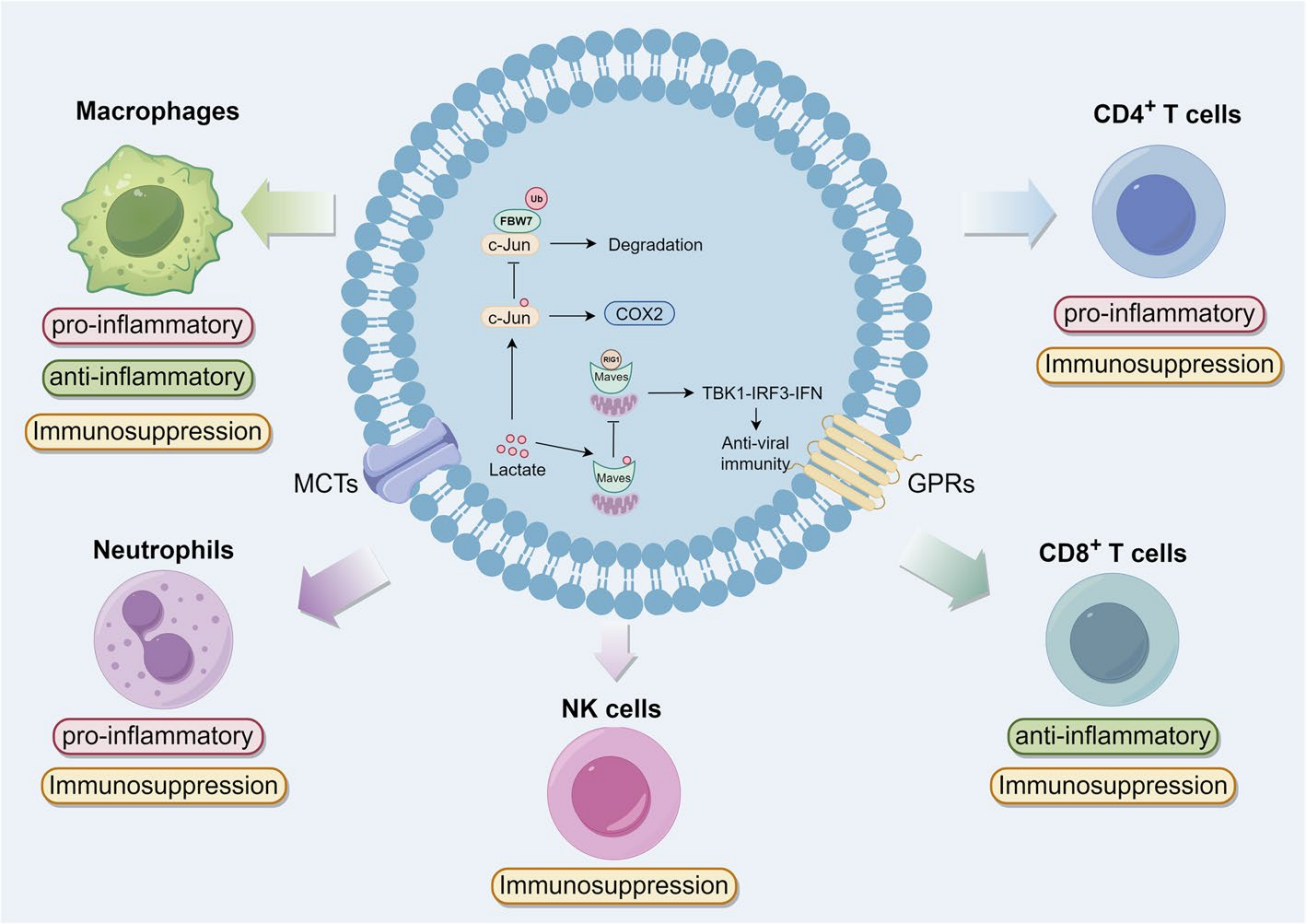
Lactate-mediated cell signaling and immune regulation. Lactate directly interacts with the transmembrane domain of MAVS, inhibiting the formation of MAVS signalosomes and consequently suppressing type I interferon production. As a novel cofactor for c-Jun, lactate prevents its degradation by the ubiquitin ligase FBW7, thereby facilitating the activation of c-Jun-driven Cox2 expression, which contributes to immune evasion. Moreover, during inflammatory activation, immune cells not only produce significant amounts of lactate via glycolysis but also detect lactate through receptors such as GPR81 or via MCTs on their surface. This lactate sensing modulates their cellular metabolism, ultimately influencing their phenotype and shifting it towards either pro-inflammatory or anti-inflammatory states

Lactate can contribute to immune evasion and facilitate the uncontrolled proliferation of tumor cells. Myeloid-derived suppressor cells (MDSCs), known for their role in promoting tumor growth and inhibiting immune responses, can be induced to accumulate by lactate through the upregulation of granulocyte–macrophage colony-stimulating factor (GM-CSF) and IL-6 [12]. In the context of pancreatic cancer radiotherapy, lactate activates the HIF-1α signaling pathway in MDSCs via the GPR81/mTOR/HIF-1α/STAT3 pathway, leading to the reprogramming of T cell responses towards an immune-suppressive phenotype, thereby promoting tumor progression [48]. Additionally, lactate has been shown to inhibit the production of IFN-γ, granzyme B, or perforin, as well as proliferation in T cells and NK cells, leading to impaired cytotoxic responses [56]. Tumor-associated neutrophils are classified into two phenotypes: anti-tumor (N1) and pro-tumor (N2). Lactate has been shown to induce polarization of neutrophils towards the N2 phenotype [57]. In immunotherapy for hepatocellular carcinoma, lactate drives the expression of programmed cell death ligand-1 (PD-L1) in infiltrating neutrophils through the MCT1/NF-κB/Cox2 pathway, thereby reducing the efficacy of immunotherapy against hepatocellular carcinoma [58] (Fig. 3). While lactate accumulation can hinder anti-tumor immune responses, it also ensures self-tolerance under physiological conditions and can prevent excessive tissue damage from pronounced immune reactions, preventing excessive tissue damage from exaggerated immune reactions. This dual role highlights lactate as a physiologically relevant metabolic checkpoint [56].

### Lactylation modification

Lysine lactylation (Kla) is a novel post-translational modification (PTM) induced by lactate, expanding our understanding of lactate's biological functions. Zhang et al. initially identified Kla in human HeLa cells and mouse bone marrow-derived macrophages (BMDMs). They identified 26 histone Kla sites in HeLa cells and 16 in BMDMs, respectively, and found that histone Kla is involved in the gene transcription of M1 macrophages [9]. Subsequent proteomic studies have confirmed the widespread nature of Kla across various cell types and tissues, and Kla also exists in various non-histone proteins. In hepatocellular carcinoma patients, 9,275 Kla sites were identified, with the majority (9,256) located on non-histone proteins, highlighting the broader scope of this modification beyond histone regulation [59]. Additionally, proteomic analysis of cardiomyocytes from heart failure mice and hypoxic astrocytes revealed 3,093 and 551 Kla sites, respectively, primarily distributed in the cytoplasm, suggesting their involvement in diverse cellular processes [60–62]. The intensity of lysine lactylation has been shown to correlate with the expression of proteins involved in lactate production and exchange. Moreover, changes in exogenous and endogenous lactate levels (such as L-lactate, sodium lactate, fisetin or 2-DG, which promotes glycolysis, or glycolytic inhibitor 2-DG) can dose-dependently regulate the levels of histone and non-histone Kla. Various factors, including hypoxia, interferon-gamma (IFN-γ), LPS or bacterial attacks can stimulate cells to produce lactate, leading to an increase in Kla levels [14].

Enzymes such as P300, SIRT2, SIRT3, HDAC2, HDAC3 and HDAC8 play crucial roles in regulating Kla. P300, a classic histone acetyltransferase, acts as a writer of histone Kla. When p300 is knocked down in mouse BMDMs, lactate-induced histone Kla is significantly reduced [63]. Yang et al. also demonstrated that specific siRNA silencing of p300 or its homolog, CREB-binding protein (CBP), attenuates lactylation modification of high-mobility group box-1 (HMGB1), indicating that p300 and its homolog CBP are potential writers of histone Kla. Collectively, these proteins work in concert to modulate the process of histone Kla modification [64]. While SIRT2, SIRT3, HDAC2, HDAC3 and HDAC8 exhibit deacetylase activity in vitro, their precise roles and mechanisms of action in regulating Kla remain to be fully elucidated [65, 66].

Lactylation plays a regulatory role in diverse physiological processes, including transcriptional activation, cellular reprogramming, fibrosis promotion, and macrophage polarization. In Alzheimer's disease (AD), elevated levels of H4K12la in glial cells adjacent to amyloid-β (Aβ) plaques are associated with enrichment and activation of transcription at the promoters of glycolytic genes, contributing to glial dysfunction [67]. During microbial sepsis conditions, macrophages can uptake extracellular lactate to promote lactylation of HMGB1, further inducing endothelial barrier dysfunction [64]. Lactate secreted by activated lung fibroblasts promotes myofibroblast differentiation, a mechanism shown to be associated with lactylation of histones in the promoter regions of pro-fibrotic genes in macrophages, facilitating the expression of pro-fibrotic mediators [63]. In ulcerative colitis, lactate contributes to the acetylation of histone H3 at lysine 9 and the lactylation of histone H3 at lysine 18, processes that promote the polarization of macrophages into the M2 phenotype [68].

Lysine lactylation also plays a crucial role in regulating gene expression and cellular function in various other contexts. For instance, H3K18 lactylation enhances the transcription of YTHDF2, which subsequently destabilizes the mRNA of key regulatory genes such as period circadian protein homolog 1 (PER1) and tumor suppressor protein p53 (TP53). This dysregulation contributes to the increased invasive proliferation and migration of ocular melanoma cells [69]. Additionally, lactate enhances the enrichment of H3K18 lactylation at the Mettl3 promoter region and directly lactylates Mettl3 at K281 and K345, thereby increasing the translation efficiency of Janus kinase 1 (Jak1) mRNA. This activation of the Jak-Stat3 pathway enhances the immunosuppressive capacity of tumor-infiltrating myeloid cells (TIMs), including macrophages, regulatory T cells and dendritic cells [66, 70].

Beyond its role in disease states, lysine lactylation also plays a significant role in normal physiological processes. Lactylation plays a crucial role in embryo development and the maintenance of endometrial receptivity. Under normal circumstances, hypoxia increases glycolysis, leading to the accumulation of endogenous lactate and subsequently increasing histone lactylation levels. In contrast, sustained hypoxia following fertilization suppresses the expression of LDHA, resulting in reduced histone lactylation levels. This inhibition ultimately hinders the developmental potential of pre-implantation embryos. Yang et al. presented the inaugural dynamic profile of lactylation in mouse oocytes and pre-implantation embryos, confirming the presence of H3K23 lactylation, H3K18 lactylation, and pan-histone lactylation throughout pre-implantation embryo development. Under hypoxic culture conditions, there was a significant increase in HIF-2α in the blastocyst-stage embryos, but a decrease in H3K23 lactylation and H3K18 lactylation. These findings, coupled with the observation that treatment with GSK2837808A effectively inhibited LDHA activity, resulting in reduced lactate production, decreased histone lactylation, and impaired embryo development [71]. Additionally, Dong et al. found that lactylation of lysine residues K228 and K232 on estrogen receptor-related receptor beta (ESRR-β) enhances its ability to promote self-renewal of embryonic stem cells (ESCs) and differentiation of extraembryonic endoderm stem cells (XEN cells), demonstrating a significant role of non-histone lactylation in embryonic development processes [72].

In addition to influencing embryonic development, histone lactylation plays a crucial role in reshaping endometrial receptivity and facilitating implantation. Yang et al. observed a significant increase in H3K18 lactylation (H3K18la) in the endometrium of pregnant ewes, while a sharp decrease in H3K18la was observed in the endometrium of failed pregnancies. These observations have led to the hypothesis of a bidirectional communication between the conceptus and the endometrium. It is suggested that, during implantation, the upregulation of glycolysis results in increased lactate production. The lactate, present in optimal concentrations at the maternal–fetal interface, may act as an embryonic-derived signal, facilitating histone lactylation modifications in the endometrium. This process plays a crucial role in modulating the redox balance, cellular apoptosis, proliferation, adhesion, and immune tolerance within the receptor endometrium [73].

The functional scope of lactylation extends far beyond these contexts, with its effects broadly encompassing multiple systemic pathological processes, including immune dysregulation, neurological disorders, and musculoskeletal injuries. Across these heterogeneous disease settings, aberrant patterns of lactylation have been shown to be closely associated with the kinetics of disease progression and responsiveness to clinical therapeutic interventions (Table 1).

**Table 1:** Impact of lactylation in diverse disease contexts

Classification	Disease	Site	Function	Reference
Gastrointestinal disorders	Colorectal cancer	H3K18	Promotes resistance to bevacizumab therapy in colorectal cancer	[74]
	Colorectal cance	CCNE2 K348	SIRT3 induces apoptosis in hepatocellular carcinoma cells by promoting de-emulsification of CCNE2	[75]
	Colorectal cance	H3K56	Drives oncogene expression and promotes tumor progression	[76]
	Gastric cancer	YAPK90, TEAD1K108	Promotes tumor cell proliferation	[77]
	Gastric cancer	METTL16K229	Trigger cuproptosis	[78]
Cardiovascular disorders	Atherosclerosis	Mecp2 k271	Inhibits atherosclerosis via the Ereg/MAPK signaling pathway	[79]
	Myocardial infarction	Snail1	Up-regulates cardiac EndoMT	[80]
	Heart failure	α-MHC K1897	Modulates the sarcomeric structure and function	[60]
Nervous system disorders	Cerebral infarction	LCP1	Promotes apoptosis and the progression of cerebral infarction	[81]
	cerebral ischemia–reperfusion	H3K18	HMGB1 induction promotes pyroptosis	[82]
	AD	H4K12	Exacerbates microglial dysfunction in AD	[67]
	AD	tau K677	Modulates iron metabolism through the MAPK pathway to drive disease progression	[83]
	Glioblastoma	XRCC1 K247	Enhances DNA repair capacity	[84]
Female Reproductive Systems	Ovarian Cancer	H4K12	Enhances DNA repair and Niraparib resistance	[85]
	Ovarian Cancer	H3K9, RAD51 K73	Facilitates homologous recombination (HR) repair and drives cisplatin resistance	[86]
	Cervical Cancer	DCBLD1 K172	Stabilizes DCBLD1 to activate the pentose phosphate pathway (PPP) and promote cancer progression	[87]
	Cervical Cancer	G6PD K45	Inhibiting lactylation activates PPP and promotes cancer cell proliferation	[88]
	Endometrial Cancer	H3K18	Upregulates USP39 to stabilize PGK1 and activate PI3K/AKT/HIF-1α signaling	[89]
	Endometriosis	H3K18	Drives ferroptosis resistance	[90]
Other disorders	Ischemic retinopathy	YY1 K183	Enhances FGF2 transcription, thereby promoting angiogenesis	[61]
	Prostate cancer	H3K18	Reconfigures chromatin accessibility and promotes lineage plasticity	[91]
	Glioblastoma	H3K9	Inhibits mismatch repair, leading to temozolomide resistance	[91]
	Psoriasis	H3K18	Promotes psoriasis progression by regulating leptin expression	[92]
	Prostate cancer	HIF1α	Promotes angiogenesis	[93]

## Lactate metabolism and lactylation: a molecular link between cellular metabolic reprogramming and multi-system disease pathogenesis

Within the intricate physiological and pathological networks of the human body, lactate metabolism and its induced protein lactylation exhibit profound systemic diversity. In the immune system, it functions as a metabolic timer that orchestrates the resolution of inflammation; meanwhile, within the tumor microenvironment, it acts as a critical driver of immune evasion and pathological angiogenesis. In the context of cardiovascular, neurological, and metabolic disorders, aberrant lactylation levels are frequently associated with the progression of tissue fibrosis and cellular dysfunction. This regulatory cascade bridging metabolic flux to alterations in protein function has emerged as a central dimension for understanding the molecular pathogenesis of multi-systemic diseases.

### Roles of lactate metabolism in cardiovascular disorders

Cardiac energy metabolism is predominantly driven by fatty acid and glucose oxidation. Under conditions of hypoxia or high-intensity workload, however, lactate can rapidly become the primary energy substrate, contributing substantially to myocardial oxidative fuel [94]. Accumulating clinical evidence demonstrates that elevated circulating lactate levels are positively associated with insulin resistance, dyslipidemia, hypertension, and overall cardiovascular metabolic risk [95–97]. Moreover, lactate serves as a robust prognostic marker for mortality and adverse outcomes in patients with acute myocardial infarction, positioning it as a potential biomarker of metabolic dysfunction in cardiovascular disease [98]. Notably, lactate exerts bidirectional effects: high concentrations promote endothelial-to-mesenchymal transition (EndoMT) following myocardial infarction, thereby aggravating myocardial dysfunction and fibrosis [80]. In contrast, exogenous lactate has been shown to be safe and capable of improving cardiac function in settings such as cardiogenic shock [99], heart failure [100], and post-coronary artery bypass grafting [101], indicating that its role extends beyond serving merely as a death signal.

MCT isoforms regulate lactate–pyruvate flux, thereby maintaining cellular energy balance and redox homeostasis [102, 103]. MCT1 mediates lactate uptake, with its membrane localization dependent on the chaperone protein CD147, serving as the primary portal for lactate entry into the heart [104]. In contrast, MCT4 has a lower affinity and is responsible for exporting glycolysis-derived lactate from cells, forming a central node of the pyruvate–lactate axis in conjunction with the mitochondrial pyruvate carrier (MPC) [105]. Loss of MCT1 results in elevated mitochondrial membrane potential, intracellular calcium and ROS accumulation, and accelerates cardiac dysfunction. Upregulation of MCT4, while alleviating intracellular lactate accumulation, promotes the development of myocardial hypertrophy and mitochondrial dysfunction [106, 107]. In diabetic cardiomyopathy, MCT4 overexpression, combined with fatty acid overload, triggers inflammation and oxidative stress. Its selective inhibitor, VB124, has been shown in animal models to reverse pathological remodeling and reduce ROS [108]. However, due to the widespread distribution of MCT4 in normal tissues, systemic inhibition may cause lactic acidosis and immunosuppression, highlighting the need for tissue-specific delivery for clinical translation. Notably, increasing exogenous lactate (sodium lactate) has been reported to improve cardiac function in patients with acute heart failure, suggesting that the direction of modulation should align with the stage of disease progression [100].

Lactylation modifications are deeply involved in the progression of various cardiac diseases. In cardiomyocytes, α-MHC-K1897la stabilizes the sarcomere and improves contractility. Inhibition of MCT4, which reduces lactate efflux, can elevate α-MHC lactylation levels and alleviate heart failure [60, 107]. Cardiac fibroblasts are activated post-ischemia, with Serpina3K-K351la exerting cardioprotective effects against ischemia–reperfusion injury via the RISK/SAFE pathways, while histone lactylation may broadly contribute to the pro-fibrotic phenotypic transition of fibroblasts and potentially play a role in cardiac fibroblast activation [109, 110]. Following myocardial infarction, monocytes rely on high glycolytic flux to supply lactate, which promotes the expression of lactylation-modified repair genes such as Lrg1, Vegf-a, and IL-10, facilitating anti-inflammatory responses and angiogenesis [111, 112]. However, lactate entering cells via MCT-dependent pathways promotes the interaction of CBP/p300 with the transcription factor Snail1 and catalyzes its lactylation. This modification enhances Snail1 activity, activates TGF-β/Smad2 signaling, and triggers EndoMT and cardiac dysfunction post-myocardial infarction [80]. In atherosclerosis, Mecp2-K271la suppresses the Ereg-MAPK axis, reducing endothelial adhesion and plaque burden[79] Tumor necrosis factor receptor–associated protein 1 (TRAP1) enhances aerobic glycolysis, elevating intracellular lactate; accumulated lactate inhibits the histone lysine delactylase HDAC3, increasing H4K12la modification, driving its transcription, inducing vascular smooth muscle cell (VSMC) senescence, and accelerating atherosclerosis [113]. In valvular disease, Lumican drives a glycolysis–H3K14/9la–Runx2/BMP2 cascade, accelerating calcific aortic valve disease [114]. In the aging heart, lactate oxidation is suppressed, the expression of lactate transporters is downregulated, and succinyl-CoA:3-oxoacid CoA transferase (SCOT) activity is increased, resulting in lactate accumulation and functional decline [112, 115, 116] In acute myocardial infarction, hyperlactatemia is closely associated with 30-day mortality, whereas heat shock protein A12A (HSPA12A) protects cardiomyocytes by maintaining H3K18la and aerobic glycolysis homeostasis [117, 118]. Moreover, lactate can promote VSMC proliferation via MCT1/4, providing an additional perspective for post-ischemic repair [119]. Collectively, lactylation fine-tunes cardiac structural and metabolic homeostasis in a cell type- and spatiotemporal-dependent manner, serving both as a marker of injury and as a potential target for precise therapeutic intervention.

### Roles of lactate metabolism in neurological disorders

The central nervous system (CNS), as one of the tissues with the highest energy demand in the body, relies on precise “metabolic coupling” between neurons and glial cells to maintain metabolic homeostasis. Lactate in the nervous system serves not only as an energy substrate, providing ATP to neurons, but also as a signaling molecule and epigenetic regulator [120]. The astrocyte–neuron lactate shuttle (ANLS) demonstrates that astrocytes convert glucose or glycogen into lactate via glycolysis and deliver it to neurons during periods of high neuronal activity, where it is oxidized to provide immediate energy for synaptic transmission [121, 122]. Notably, under certain neuropathological conditions, a tumor-like “aerobic glycolysis” (Warburg effect) can be recapitulated, leading to abnormal lactate accumulation and contributing to the metabolic reprogramming associated with neurodegeneration [123].

The dual regulatory roles of lactate metabolism in the central nervous system (CNS) have been substantiated by extensive experimental evidence. Lactate provides carbon sources for neuronal oxidative phosphorylation through both the astrocyte–neuron lactate shuttle (ANLS) and the intracellular mitochondrial lactate oxidation complex (mLOC), while coupling with fatty acid synthesis to sustain ATP and membrane precursor supply required for synaptic plasticity [122, 124, 125]. Pathologically, increased lactate in neurons promotes reactive oxygen species (ROS) production, inducing oxidative stress, impairing ATP synthesis, further enhancing mitochondrial ROS generation, and leading to peripheral nervous system mutations that inflict significant damage on the CNS [126]. Lactate also acts as an endogenous ligand for the postsynaptically enriched G-protein-coupled receptor GPR81, regulating angiogenesis, cerebral blood flow, and metabolic reprogramming of the tumor microenvironment, while promoting learning and memory formation via the SIRT1–BDNF pathway [127]. In Alzheimer’s disease models, decreased lactate levels are significantly negatively correlated with cognitive decline, and exogenous lactate supplementation can reverse memory deficits induced by MCT2 downregulation, suggesting that targeting lactate metabolism may represent a potential therapeutic strategy for neurodegenerative diseases and neural injury [128].

Lactylation modifications have emerged as central epigenetic nodes in elucidating the pathological progression of neurological disorders. In Alzheimer’s disease (AD), glycolytic bursts in microglia lead to lactate accumulation, which enriches H4K12la at promoters of glycolytic genes and forms a positive feedback loop with PKM2, driving pro-inflammatory activation and exacerbating metabolic dysregulation and microglial dysfunction in AD patients [67]. Following spinal cord injury, H3K27la activates the transcription factor Olig2, promoting the differentiation of oligodendrocyte precursor cells (OPCs) into mature oligodendrocytes and facilitating remyelination, thereby contributing to motor recovery [129]. In the aging brain, H3K18la targets the promoters of RelA/NFκB1, reinforcing the senescence-associated secretory phenotype (SASP) and accelerating brain aging and AD progression [130]. After ischemic stroke, MeCP2-K210la drives microglial activation via the HK2/mTOR axis, promoting mitochondrial dysfunction and sustaining inflammatory activation and pathological proliferation of microglia [131]. However, Sun et al. demonstrated that lactylation at MeCP2 K210/K249 during ischemic brain injury suppresses the expression of apoptosis-related genes, including programmed cell death protein 4 (Pdcd4) and phospholipase A2 group VI (Pla2g6), thereby reducing neuronal apoptosis [132]. Collectively, both histone and non-histone lactylation regulate neuroinflammation, energy metabolism, and cell fate in a cell type- and stimulus-dependent manner, suggesting that targeting lactate production or the “writing–erasing” machinery of lactylation may offer potential therapeutic strategies for neurological disorders.

### Roles of lactate metabolism in autoimmune disorders

Metabolic reprogramming of immune responses has been recognized as a central determinant of immune cell fate and function, with glycolysis-derived lactate and its mediated lysine lactylation rapidly emerging as key molecular hubs linking immune metabolic dysregulation to autoimmune pathology. Lactate is now redefined as a signaling molecule with dual immunomodulatory properties: it can both enhance the immunosuppressive function of regulatory T cells and synergistically drive Th17-mediated pro-inflammatory responses, revealing its bidirectional antagonistic role in immune homeostasis regulation [133]. Elevated lactate accumulation and globally increased protein lactylation are observed in multiple autoimmune diseases, with their magnitude positively correlating with disease activity [134]. Increased lactate levels promote inflammatory responses in critical immune cell subsets, including dendritic cells, macrophages, and T cells, which constitute the core mediators of autoimmune pathology [135]. In autoimmune diseases, immune cell metabolic reprogramming regulates lactylation levels via glycolytic byproducts, while lactylation, in turn, modulates cellular metabolism through conformational remodeling and transcriptional reprogramming, forming a metabolism–epigenetic positive feedback loop that synergistically accelerates disease progression.

Lactate and its mediated lactylation modifications play complex and pivotal roles in immune system diseases. In rheumatoid arthritis (RA), lactate accumulates in synovial cells, serving as a potential disease biomarker [136]. Enhanced aerobic glycolysis in synovial cells and activated T cells upregulates lactate dehydrogenase A (LDHA) expression, which promotes interferon-γ (IFN-γ) secretion and histone acetylation, thereby sustaining effector T cell function [137]. Elevated extracellular lactate is taken up via SLC5A12 (SMCT2) in CD4⁺ T cells and MCT1 in CD8⁺ T cells, restricting T cell migratory capacity, causing retention within synovial fluid, and driving CD4⁺ T cell differentiation into Th17 cells with IL-17 production, while impairing the cytolytic function of CD8⁺ T cells [53, 138]. Synovial fibroblasts (RASFs) export lactate via MCT4, exacerbating synovial acidification; targeting MCT4 silencing in animal models alleviates arthritis severity [138]. Additionally, lactylation modifications regulate disease progression, for instance, artesunate promotes PKM2 lactylation to inhibit its nuclear translocation, thereby attenuating synovial hyperplasia [139]. In ulcerative colitis (UC), lactate exerts context-dependent effects. Lactate produced by the gut microbiota, such as Bifidobacterium and Lactobacillus, signals through GPR81 to suppress dendritic cell and macrophage-mediated inflammation, promotes regulatory T cell (Treg) induction, and limits Th1/Th17 responses [140]. Rectal administration of lactate in mouse models alleviates colitis, potentially by reducing IL-6 levels, inhibiting NLRP3 inflammasome activation via MCT-mediated macrophage lactate uptake, and inducing histone H3K18 lactylation [141]. However, in human UC, dysbiosis-induced lactate overaccumulation correlates with disease exacerbation, possibly by creating a pro-inflammatory microenvironment, disrupting short-chain fatty acid balance, and impairing epithelial barrier function [142]. In allergic diseases such as asthma, plasma lactate levels correlate with disease severity. Th2 and Th17 cells in bronchoalveolar lavage fluid overproduce IL-4, inducing glucocorticoid resistance, a process potentially reinforced by lactate [143]. Conversely, lactate stimulation can suppress IgE and IL-33 production by mast cells, suggesting a potential role in mitigating allergic responses [144].

Furthermore, lactate metabolism and lactylation are critical in other autoimmune diseases. In systemic lupus erythematosus (SLE), enhanced glycolysis and lactate accumulation drive immune cell activation, with lactate promoting cGAS protein lactylation and modulating type I interferon responses [145]. In primary Sjögren’s syndrome (pSS), elevated lactate in salivary glands induces mitochondrial DNA damage, activating the cGAS–STING pathway and inflammatory responses [146]. In multiple sclerosis (MS), increased lactate levels in the central nervous system correlate with disease activity and promote Th17 differentiation [53]. In psoriasis, lactate accumulation in the skin stabilizes RORγt to enhance Th17 responses and may regulate gene expression via histone lactylation [147, 148]. In systemic sclerosis (SSc) and autoimmune uveitis, lactate metabolic reprogramming and lactylation modifications contribute to fibrosis, microglial activation, and Th17 differentiation, respectively, participating in disease pathogenesis [149–151].

In summary, lactate not only influences immune cell function, differentiation, and migration as a metabolic byproduct, but lactate-mediated lactylation also acts as a critical epigenetic mechanism, broadly regulating the expression of immune-related genes and signaling pathways. Together, these processes play central roles in the pathogenesis of diverse immune system diseases. The ultimate effects of lactate and lactylation are determined by complex factors, including local concentration, cellular origin, microenvironment, and overall immune metabolic state.

### Roles of lactate metabolism in other diseases

Lactate and its mediated lysine lactylation modifications are widely involved in the onset and progression of various systemic diseases, serving as key epigenetic and post-translational mechanisms linking cellular metabolic states to pathological processes.In respiratory diseases, airway inflammation in asthma is closely associated with metabolic reprogramming. The corticosteroid dexamethasone exerts therapeutic effects by suppressing the Hif-1α–glycolysis axis, thereby downregulating protein lactylation [152]. In lung cancer, lactylation drives tumor progression and immune evasion through multiple mechanisms: H3K18la enrichment promotes the expression of genes such as IDH3G, accelerating proliferation and migration of lung adenocarcinoma cells [153]; H4K8/H4K16 lactylation activates TERT transcription via Sp1, maintaining telomere length [154]; hypoxia-induced SOX9 lactylation enhances tumor stemness[155]. Lactylation also contributes to therapy resistance by promoting transcription of neuroendocrine differentiation-associated genes, accelerating the cell cycle via H4K12la, and stabilizing APOC2 protein expression through lactylation, which facilitates regulatory T cell accumulation and immune therapy resistance [156]. In the hypoxic environment of malignant pleural effusions, lactate upregulates TNFR2 expression in Treg cells via H3K18la, enhancing their immunosuppressive function [157]. In pulmonary fibrosis, the key process of fibroblast-to-myofibroblast transition is directly driven by lactylation. Silica exposure or TGF-β1 stimulation increases H3K18la and H4K12la levels in lung tissue, enriching these modifications at promoters of pro-fibrotic genes and promoting α-SMA and collagen I expression [158]. Lactate produced by myofibroblasts can also enter alveolar epithelial cells via MCT1, inducing YTHDF1 transcription and establishing a pro-fibrotic positive feedback loop [159].

In the field of metabolic diseases, lactylation represents a key regulatory node. In diabetic nephropathy, the number of lactylation sites on renal proteins is significantly increased, with K182 lactylation of ACSF2 leading to mitochondrial dysfunction [160]. Histone lactylation also promotes epithelial–mesenchymal transition by upregulating KLF5 expression [161]. Regarding diabetic complications, lactylation drives alterations in adipose tissue mass and obesity-related proteins, contributing to angiogenesis in diabetic retinopathy [162]. In gestational diabetes, elevated systemic lactylation levels are associated with key regulatory proteins such as CACNA2D1 [163]. In diabetic cardiomyopathy, lactate accumulation enhances H4K12 lactylation via MCT4, inducing inflammatory infiltration [108].

In digestive system diseases, lactylation drives disease progression through metabolic–epigenetic crosstalk. In esophageal cancer, H3K9la acts as an activating mark to promote transcription of genes such as LAMC2, enhancing tumor migration and invasion [164]. Under hypoxic conditions, lactylation of SHMT2 enhances its enzymatic activity, sustaining tumor stemness and chemoresistance [165]. In colorectal cancer, hypoxia-induced lactylation, such as H3K18la, maintains cancer stem cell properties by activating the Wnt/β-catenin signaling pathway and induces autophagy through upregulation of RUBCNL, contributing to bevacizumab resistance [74]. Hepatocellular carcinoma exhibits widespread non-histone lactylation modifications, affecting glycolysis, the tricarboxylic acid cycle, mitochondrial function, and other metabolic pathways. Lactylation also regulates regulatory T cell function, promoting an immunosuppressive microenvironment [166]. In pancreatic cancer, NUSAP1 forms a positive feedback loop with LDHA to sustain glycolysis and lactate production [167, 168]. Accumulated lactate in the pancreatic tumor microenvironment can enter CD8⁺ T cells via MCT1, suppressing their glycolytic activity and effector function, thereby mediating immune evasion [167].

In kidney diseases, lactylation modifications represent key mechanisms underlying both acute and chronic injury. During acute kidney injury, lactate levels are inversely correlated with renal function, and its accumulation induces mitochondrial dysfunction in renal tubular epithelial cells [169, 170]. In sepsis-induced injury, lactate mediates K20 lactylation of mitochondrial fission protein 1 (Fis1) and K52 lactylation of ALDH2, which respectively exacerbate mitochondrial hyperfission and inhibit mitophagy, thereby aggravating tubular damage [171]. Moreover, the progression of chronic kidney disease involves fibrosis, inflammation, and metabolic dysregulation, with lactylation contributing through modulation of inflammatory responses and related processes [170].

In summary, lactate and lactylation modifications, as ubiquitous metabolic and epigenetic regulatory mechanisms, play central roles across a broad spectrum of systemic diseases, including female reproductive disorders, neurological diseases, cardiovascular diseases, immune system disorders, respiratory diseases, digestive diseases, and kidney diseases. By modulating gene transcription, protein function, the immune microenvironment, and cell fate decisions, they profoundly influence disease initiation, progression, and therapeutic responses, offering novel perspectives and potential targets for diagnosis and treatment.

## The female reproductive system: an exceptional paradigm integrating cyclical tissue remodeling, transgenerational programming, and pathological plasticity via lactylation

Within the research landscape of pan-systemic disease mechanisms, the female reproductive system constitutes a core paradigm for studying lactate metabolism and lactylation due to its unparalleled physiological complexity. Distinguishable from the relatively constant biological background of other tissues, this system undergoes recurrent cycles of tissue shedding and regeneration, where such highly dynamic periodic metabolic remodeling provides an exceptional model for observing how lactate functions as a signaling hub to coordinate physiological inflammation and tissue repair. Furthermore, the naturally hyper-lactatemic state and low-pH niche of the reproductive tract microenvironment establish extreme and unique metabolic sensing mechanisms, rendering the role of lactylation in regulating host immune tolerance, embryo implantation, and pathological fibrosis far more representative than in other systems. Additionally, lactylation levels within the reproductive system are directly linked to germ cell quality and the establishment of early epigenetic programs, with its regulatory significance in transgenerational metabolic memory endowing this research with profound implications that transcend individual pathogenesis. Consequently, an in-depth exploration of lactate-driven mechanisms in the female reproductive system not only elucidates the underlying logic of gynecological malignancies and infertility but also provides a decisive scholarly contribution to the global mapping of human diseases regarding the metabolic regulation of complex life-cycle evolution.

### Ovarian cancer: metabolic reprogramming and microenvironmental acidification driving malignant progression and epigenetic memory

Ovarian cancer (OC) is the deadliest gynecological malignancy, responsible for an estimated 140,000 deaths worldwide annually. As reported by the International Agency for Research on Cancer in 2020, OC ranked 18th in terms of newly diagnosed cases and 14th in terms of mortality worldwide [172]. Therefore, elucidating the molecular mechanisms underlying the occurrence and progression of OC is crucial for designing novel therapeutic strategies to improve patient prognosis.

The prevalence of aerobic glycolysis in ovarian cancer has been confirmed in both clinical specimens and preclinical models. Compared to early-stage (I/II) cases, late-stage (III/IV) ovarian cancer exhibits significantly elevated levels of glycolytic enzymes, which are often associated with metastasis [173]. Liu et al. demonstrated a strong correlation between enhanced glycolytic activity and poor prognosis in ovarian cancer patients [174].

Hexokinase 2 (HK2), a key rate-limiting enzyme in glycolysis, plays a pivotal role in regulating lactate production, promoting ovarian cancer metastasis and stemness through the FAK/ERK1/2 signaling pathway-mediated MMP9/NANOG/SOX9 expression [175]. The expression of HK2 is regulated by a variety of signaling pathways and transcription factors, such as PI3K/AKT, FAK/ERK1/2, RAS, HIF-1 and STAT2. MiR-145 directly targets and inhibits the expression of HK2, while also indirectly downregulating the levels of PKM2 and LDH, leading to a reduction in glucose utilization and lactate production. DNA methyltransferase 3 A (DNMT3A) enhances glucose uptake and lactate production by upregulating the expression of HK2 and PKM2. As a negative regulator of the Warburg effect induced by DNMT3A in ovarian cancer cells, MiR-145 emerges as a promising therapeutic target for enhancing anticancer treatments [176].

Phosphofructokinase 1 (PFK1) catalyzes the conversion of fructose-6-phosphate to fructose-1,6-bisphosphate, an irreversible reaction that plays a critical role in controlling the rate and extent of glycolysis. Elevated PFK activity has been linked to poor metastasis and survival rates. Endogenous nitric oxide synthase NOS1 can stabilize PFKM tetramerization by inducing S-nitrosylation at the Cys351 site of PFKM, thus counteracting negative feedback from downstream metabolites, resulting in increased glycolysis and tricarboxylic acid cycle flux [177]. CXCL14 is a secretory pro-mediator produced by cancer-associated fibroblasts (CAFs), which induces the binding of long non-coding RNA (LINC RNA) 00092 to PFKFB2 to promote ovarian cancer (OC) metastasis [178].

Pyruvate kinase (PKM), the terminal rate-limiting enzyme in glycolysis, facilitates the conversion of phosphoenolpyruvate to pyruvate, a reaction that is coupled with the production of ATP. Research indicates that PSMD14 promotes the deubiquitination of PKM2, leading to increased dimerization and nuclear translocation, enhancing the transcription of downstream oncogenes, and stimulating the malignant progression of ovarian cancer [179]. Furthermore, AXL, AKT2, and FSH directly regulate the expression of PKM2, thereby enhancing aerobic glycolysis. This modulation subsequently drives increased cell proliferation, invasion, migration, and resistance to cisplatin in ovarian cancer cells [180–182]. Conversely, CHIP can promote the ubiquitination and degradation of PKM2, downregulating its expression and reducing glycolysis and lactate production [183].

LDHA is the enzyme catalyzing the final step of the glycolytic pathway, converting pyruvate and NADH to L-lactate and NAD. It has been reported that LDHA is upregulated in ovarian cancer compared to normal tissue [184]. As a tumor suppressor gene, E3 ubiquitin ligase tripartite motif-containing 3 (TRIM3) directly interacts with LDHA, promoting its ubiquitination and degradation. This process inhibits glycolysis and suppresses lactate production in ovarian cancer cells [185]. Furthermore, miR-383 regulates the expression of LDHA in ovarian cancer cells, thereby impeding aerobic glycolysis, cell proliferation, and invasion [186].

When cancer cells grow to form tumor masses, the oxygen concentration decreases at the center, causing cells to become hypoxic and activating hypoxia-inducible factors [187]. Hypoxia-inducible factors are transcription factors located in the cell nucleus that facilitate cellular adaptation to low-oxygen conditions. These include HIF-1α, HIF-2α, and HIF-3α. In ovarian cancer, HIF-1α is often overexpressed and plays a crucial role in regulating the transcription of multiple target genes, such as erythropoietin, GLUT1, HK2, PFK1, PKM, and VEGF. Through this regulation, HIF-1α enhances glucose uptake and lactate production, contributing to the metabolic reprogramming characteristic of cancer cells. Additionally, lactate has been shown to stabilize HIF-1α in the tumor microenvironment, promoting angiogenesis and immune evasion, thereby further advancing cancer progression through this positive feedback loop. Cryptotanshinone inhibits glucose uptake and lactate production induced by cell glycolysis by suppressing the STAT/SIRT3/HIF-1α signaling pathway, thereby inhibiting cell growth and proliferation induced by cell glycolysis [188]. The HIF-1α inhibitor EF24 reverses the glycolytic effect in OC by downregulating GLUT-1 expression, resulting in significantly reduced glucose uptake, glycolytic rates, and lactate production. This inhibition suppresses the proliferation, invasion, and metastasis of ovarian cancer cells [189]. Additionally, c-Myc can synergistically interact with HIF-1 on pyruvate dehydrogenase kinase 1 (PDK1), promoting the conversion of glucose to lactate. Knockdown of fructose-1,6-bisphosphatase 1 (FBP1) significantly reverses the low expression of c-Myc, increasing glucose consumption and lactate production, thereby promoting the progression and cisplatin resistance of ovarian cancer [190].

**Fig. 4:**
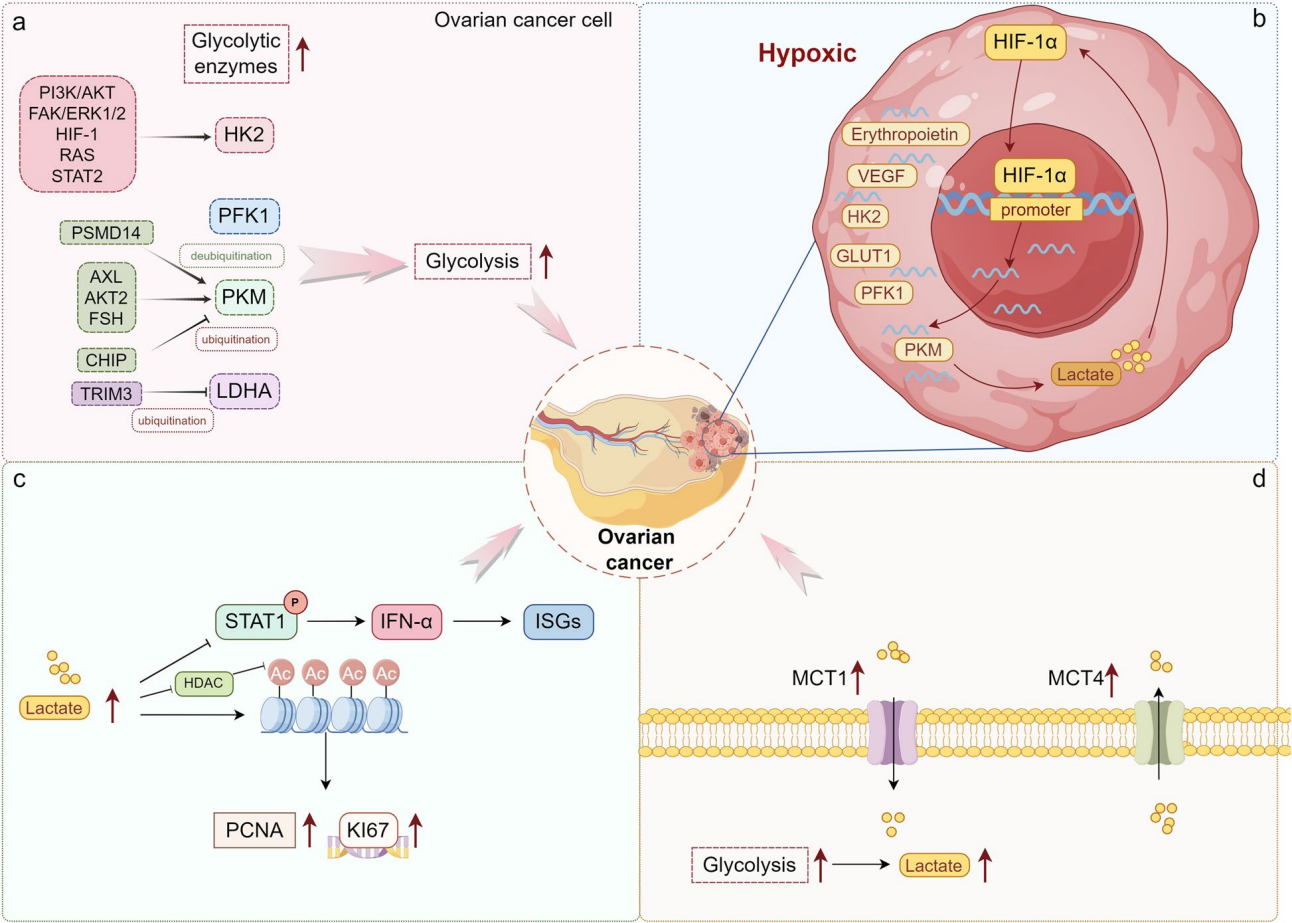
Mechanisms of lactate metabolism in ovarian cancer. **a**. Key rate-limiting enzymes in glycolytic pathways, including HK2, PFK1, PKM, and LDHA, are upregulated in ovarian cancer, promoting glycolysis and lactate production, thus facilitating tumor cell proliferation and invasion. **b**. Decreased oxygen levels in the tumor core place cancer cells in hypoxic conditions, activating HIF-1α, which is overexpressed and regulates the transcription of various genes, including erythropoietin, GLUT1, HK2, PFK1, PKM, and VEGF, promoting glucose uptake and lactate production. Lactate stabilizes HIF-1α in the stroma, enhancing angiogenesis and immune evasion, forming a positive feedback loop. **c**. Lactate decreases the levels and phosphorylation of STAT1 protein in ovarian cancer cells, inhibiting IFNα-induced ISG expression and ultimately suppressing antitumor activity. Additionally, lactate promotes HDAC inhibition and overall histone acetylation, ultimately enhancing ovarian cancer cell proliferation by upregulating PCNA and KI67 genes. d. MCT1 and MCT4 facilitate rapid lactate transport in ovarian cancer cells, participating in disease progression

The increase in glycolysis not only meets the greater metabolic energy demands of tumors but also leads to acute and chronic acidification of the local environment through the conversion of pyruvate to lactate. This microenvironmental acidosis inhibits gap junction conductivity, thereby suppressing invasion and metastasis, and may activate matrix metalloproteinases that promote extracellular matrix and basement membrane degradation [189, 191]. Dong et al. found that lactate can reduce the protein levels and phosphorylation of STAT1 in ovarian cancer cells, thereby inhibiting interferon-α (IFN-α) induced expression of interferon-stimulated genes (ISGs) and ultimately suppressing antitumor activity. They also demonstrated that LDHA knockdown combined with IFN-α treatment effectively enhances the anti-ovarian tumor response, such as increased infiltration and activity of CD8 + T cytotoxic cells and NKT cells, providing new insights for improving the efficacy of IFN-α treatment [192]. Bandopadhyay et al. found that paired-like homeodomain transcription factor 2 (PITX2) induces protein kinase B (AKT)-mediated glycolysis in ovarian cancer cells. Overexpression of PITX2 promotes the nuclear translocation of LDHA and enhances lactate production. Lactate promotes histone deacetylase (HDAC) inhibition and global histone acetylation. This may ultimately lead to increased proliferation of ovarian cancer cells by upregulating the proliferating cell nuclear antigen (PCNA) and KI67 genes [193]. Of note, research has found higher levels of MCT1 and MCT4 expression in epithelial ovarian cancer (EOC) cell lines, primary EOC tissues, and metastatic lesions compared to benign and normal ovarian tissues. These transporters play a crucial role in the rapid transport of lactate, essential for the heightened glycolysis in tumor cells. The study also demonstrated that elevated levels of MCT1/MCT4 are associated with tumor grade, clinical stage, residual tumor, and ascites, and are involved in the progression and metastasis of EOC, potentially serving as a target for controlling EOC metastasis [194] (Fig. 4).

### Cervical cancer: interplay between viral oncogenes, microbiota-derived lactate, and host immune plasticity

Cervical cancer remains a significant global health burden, ranking fourth in both incidence and mortality rates. In 2020, there were 342,000 deaths and 604,000 new cases reported [195]. As a typical feature of cancer cells, enhanced aerobic glycolysis and increased lactate production exist in cervical cancer, which are significantly associated with tumor recurrence and metastatic potential, ultimately leading to poor patient prognosis. Chen et al. found the NAT10/ac4C/FOXP1 axis as a driver of glycolysis and lactate production in cervical cancer, and the lactate-rich tumor microenvironment (TME) further promotes the immunosuppressive characteristics of tumor-infiltrating regulatory T cells (Tregs) [196]. Additionally, HPV E6/E7 activates insulin-like growth factor 2 mRNA-binding protein 2 (IGF2BP2) to induce m6A methylation modification of c-Myc mRNA, further promoting aerobic glycolysis, proliferation, and metastasis of cervical cancer [197]. Beyond its role in histone modifications, non-histone lactylation modification has also been implicated in the occurrence and development of cervical cancer. Meng et al. confirmed that HPV-16 E6 promotes the formation of G6PD dimers and increases its enzyme activity by inhibiting lactylation of the K45 site of glucose-6-phosphate dehydrogenase (G6PD), thereby activating the pentose phosphate pathway (PPP) to promote proliferation of cervical cancer cells [88]. Furthermore, L-lactate inhibits the degradation of DCBLD1 by directly increasing lactylation modification of DCBLD1, suppressing G6PD autophagic degradation and activating PPP, ultimately promoting cervical cancer progression [87]. Xiao et al. found that lactate stimulation promotes the translocation of β-catenin from the cytoplasmic membrane to the nucleus, which in turn triggers the redistribution of fascin between the nucleus and cytoplasm, specifically to the protrusion compartment. Moreover, in both in vitro and in vivo murine xenograft models, the application of a lactate antagonist effectively inhibited lactate-induced nuclear import of β-catenin, nuclear export of fascin, and the growth and invasive behavior of cervical cancer cells [198]. Ciszewski et al. proposed that lactate enhances hydroxycarboxylic acid receptor 1 (HCA1) signaling and induces transcription of DNA repair genes, as well as recruitment of DNA-PKcs, breast cancer susceptibility gene 1 (BRCA1), and nibrin to the nuclear compartment, due to lactate's inhibitory activity on histone deacetylases, leading to chromatin acetylation. The lactate-enriched microenvironment enhances the repair of genomic DNA damage induced by chemotherapy or radiation, thereby promoting the survival of cancer cells. The upregulation of cellular DNA repair mechanisms is a critical factor that contributes to the resistance of cervical cancer to radiotherapy and chemotherapeutic treatments [199]. Additionally, extracellular lactate supplementation reduces the expression of HPV-16 E6 and E7 oncogenes and promotes migration and invasion of SiHa cervical cancer cells through upregulation of the miR-774/ARHGAP5 axis [200]. Inhibition of lactate production or transport decreases the expression of M2 macrophage markers in macrophages co-cultured with HPV-positive cell lines, while also enhancing the ability of these macrophages to activate T lymphocytes [201]. In summary, targeting lactate metabolism may be an effective strategy for anticancer therapy in cervical cancer. Therefore, aberrantly expressed lactate transporters MCTs and GPR81 in cervical cancer merit further investigation.

**Fig. 5:**
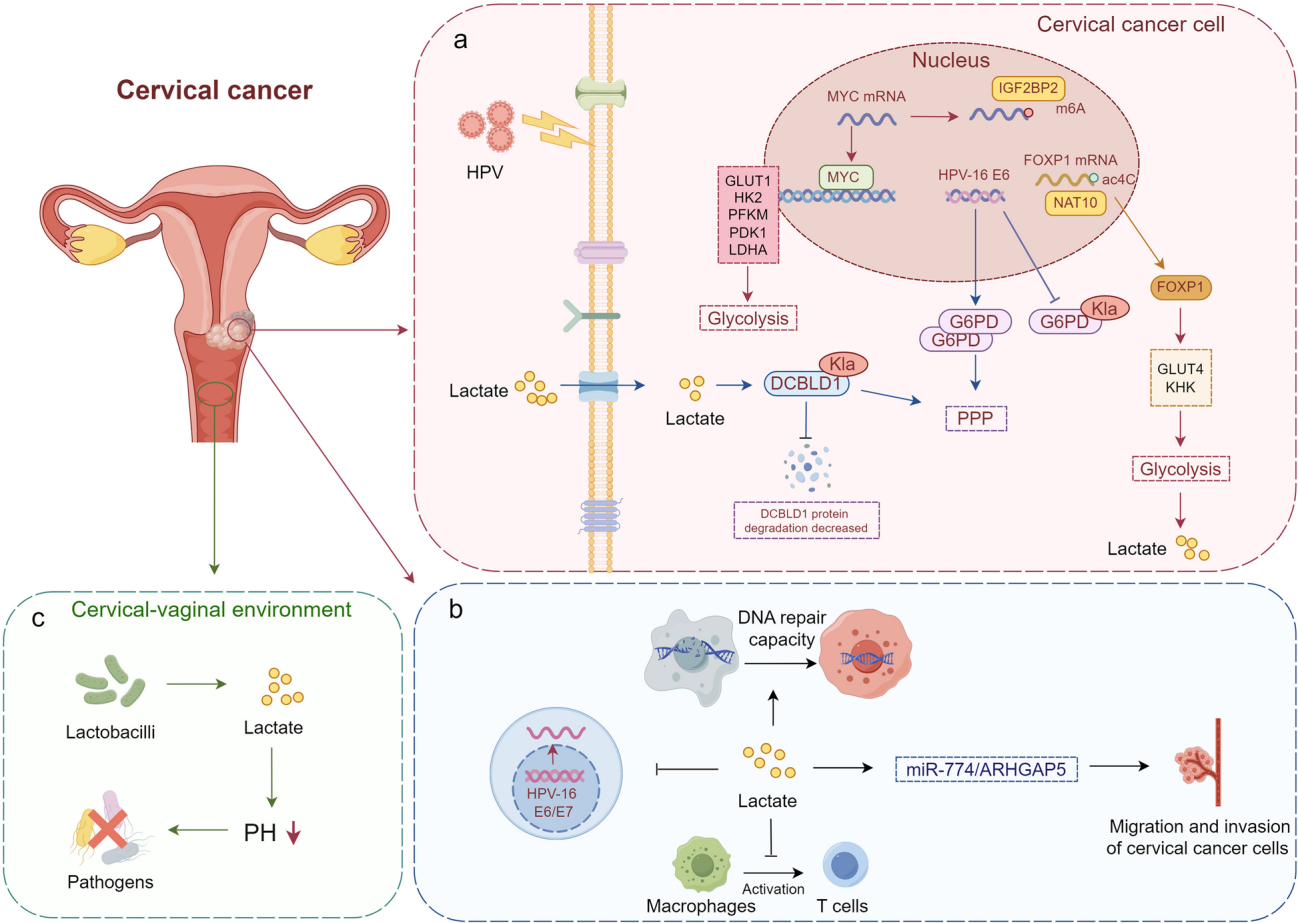
Mechanisms of lactate metabolism in cervical cancer. **a**. PPP and glycolytic pathway are upregulated in cervical cancer, promoting tumor progression. **b**. Upregulated lactate in tumor cells accelerates the repair of genomic DNA damaged by anticancer drugs/radiation, promoting cancer cell survival. Lactate decreases the expression of HPV-16 E6 and E7 oncogenes, but simultaneously promotes the migration and invasion of cervical cancer cells by upregulating the miR-774/ARHGAP5 axis. Moreover, inhibition of lactate synthesis or transport improves the activation potential of T lymphocytes in macrophages. **c**. Lactate in the cervical-vaginal environment is primarily produced by lactobacilli, which lowers the local pH and creates an inhospitable environment for pathogens, thereby helping to prevent infectious diseases

The female lower reproductive tract is characterized by high lactate concentrations, with vaginal secretions containing up to 50 mM of lactate [202]. In addition to glycolysis, the main source of lactate in the cervical-vaginal environment is derived from lactobacilli. Lactobacilli promote cervical-vaginal health by producing lactate and other antibacterial compounds. Lactate effectively lowers local pH and creates an unfavorable environment for pathogens, thereby preventing pathogenic diseases such as urinary tract infections, sexually transmitted infections, and bacterial vaginosis [203]. The lactate subtypes secreted by Lactobacillus species such as L. crispatus, L. gasseri, and L. jensenii are protective factors against HPV infection and cervical lesions [204]. Numerous studies indicate a close correlation between the absence of lactobacilli in the female reproductive tract and persistent high-risk HPV infection and tumor progression. However, Colbert et al. discovered that inert lactobacilli, such as L. iners, in the tumor microenvironment may have a symbiotic relationship with cancer cells. L. iners, a facultative anaerobe, can generate ATP through aerobic respiration and efficiently produce lactate under anaerobic conditions. It has been linked to reduced patient survival, the induction of chemoradiotherapy resistance in cervical cancer cells, and the reprogramming or alteration of various metabolic pathways within tumors [205] (Fig. 5).

### Endometrial cancer: the feedback loop linking glycolytic surge to histone lactylation and invasive phenotypes

Endometrial cancer (EC) is the sixth most common cancer in women and one of the three most common malignancies in gynecology. Its incidence has been steadily increasing worldwide [206]. Elevated lactate levels in both serum and lesion tissues of endometrial cancer patients have been confirmed by multiple studies, consistent with the higher glycolytic rate in cancer cell metabolism [207, 208]. Wang et al. demonstrated that small nucleolar RNA host gene 9 (SNHG9) directly increases the expression of HK2, which subsequently activates the FAK/ERK1/2 signaling pathway. This activation promotes aerobic glycolysis in endometrial cancer cells, driving cell proliferation and facilitating invasive growth via epithelial-mesenchymal transition (EMT) [209]. The elevated expression of PKM2 is strongly linked to poor prognosis in endometrial cancer. Estrogen induces the upregulation of PKM expression through the c-Myc/hnRNP splicing pathway, facilitating the formation of PKM2. This results in the disruption of its tetrameric structure and triggers its translocation to the nucleus, thereby promoting the Warburg effect [210]. Gong et al. identified that anterior gradient 2 (AGR2) interacts with MUC1 to stimulate the expression of HIF-1α. In turn, HIF-1α regulates key proteins such as LDHA, HK2, ENO1, and PGK1, driving a metabolic shift from oxidative phosphorylation to glycolysis. This shift results in increased lactate production, enhanced vascularization, and the promotion of an invasive phenotype in endometrial cancer [211]. Additionally, kinesin family member 23 (KIF23) is upregulated under hypoxic conditions in a HIF-1α-dependent manner and participates in lactate metabolism in uterine corpus endometrial carcinoma (UCEC) by regulating LDHA expression [212]. Shi et al. constructed a prognostic model based on lactate metabolism-related genes (LMRGs) and further identified translocase of inner mitochondrial membrane 50 (TIMM50) as a key potential prognostic marker for EC. Experimental evidence confirmed that TIMM50 promotes proliferation, migration, and lactate generation in EC cells [213].

**Fig. 6:**
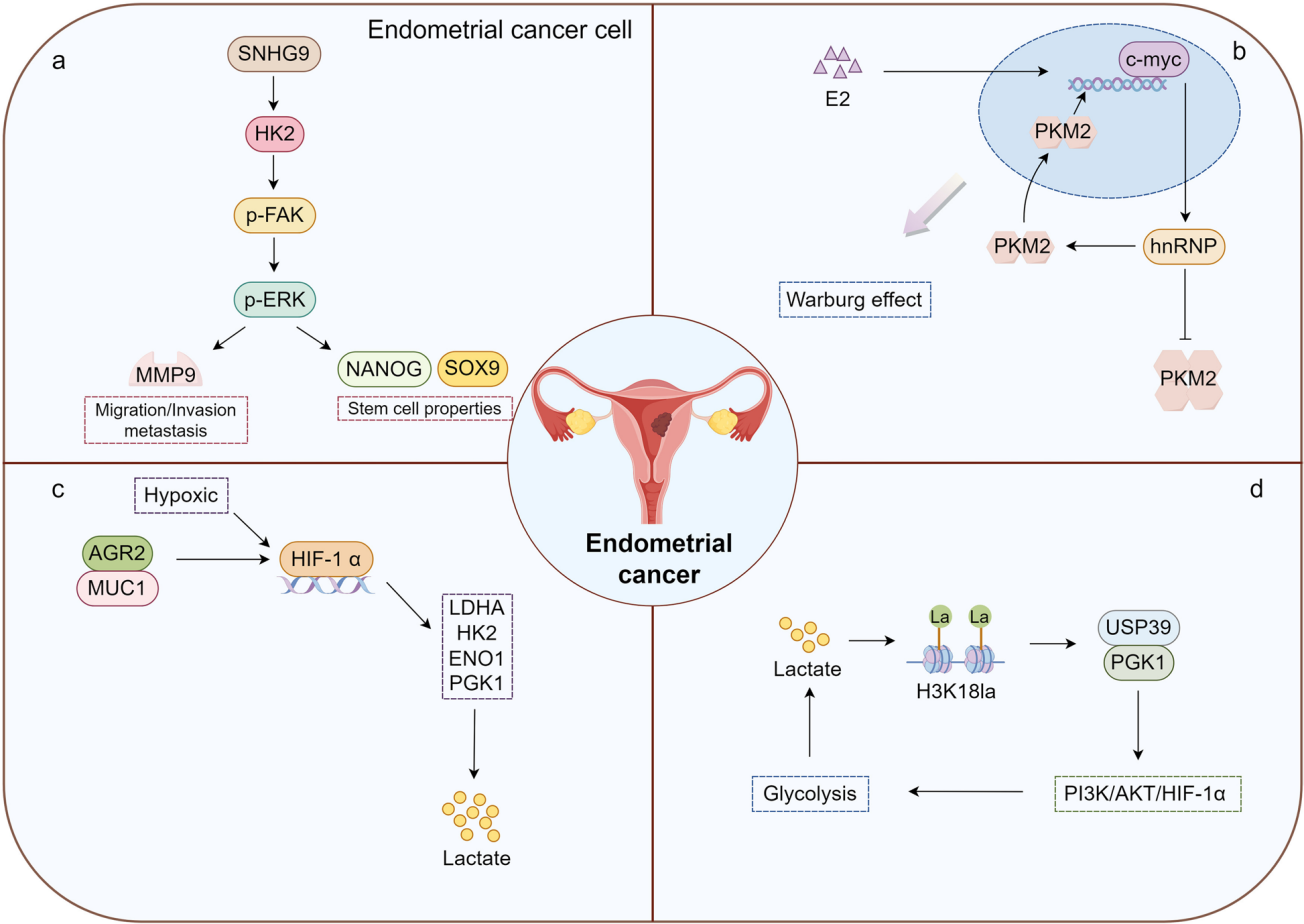
Mechanisms of lactate metabolism in endometrial cancer. **a**. SNHG9 directly upregulates HK2 expression, leading to HK2 overexpression of FAK and its downstream ERK1/2 signaling pathways, enhancing cell migration, invasion, and stemness regulation in tumor cells. **b**. Estradiol upregulates PKM expression via c-Myc/hnRNP, promoting the formation of PKM2, which disrupts its tetrameric structure and promotes nuclear translocation, thereby inducing the Warburg effect. **c**. AGR2 interacts with MUC1 to activate the expression of HIF-1α, which in turn regulates critical glycolytic enzymes such as ALDH, HK2, ENO1, and PGK1. This shift from oxidative phosphorylation to glycolysis enhances lactate production, while simultaneously promoting angiogenesis and the development of invasive phenotypes. **d**. Lactate stimulates lactylation of histone H3 at lysine 18 in endometrial cancer, promoting interaction, stabilization, and deubiquitination of USP39 and PGK1, thereby activating the PI3K/AKT/HIF-1α signaling pathway, stimulating glycolysis and producing more lactate, creating a positive feedback loop

Many studies have confirmed the enhanced glycolytic activity in EC, creating favorable conditions for cancer cell proliferation, invasion, and migration, along with a concurrent increase in lactate, the primary product of glycolysis [214–217] While the mechanisms underlying lactate's actions in EC remain to be fully elucidated. Recent research has revealed that excess lactate produced in EC stimulates lactylation of histone H3 lysine 18, regulating the expression of ubiquitin-specific peptidase 39 (USP39). USP39, through interaction with PGK1, activates the PI3K/AKT/HIF-1α signaling pathway, thereby stimulating glycolysis to produce more lactate, further increasing histone lactylation. This positive feedback loop further promotes the growth and metastasis of EC [89]. Given the critical involvement of lactate in tumor initiation and progression, targeting lactate metabolism offers a promising strategy for further research and potential therapeutic interventions in endometrial cancer (Fig. 6).

### Endometriosis and adenomyosis: lactate-mediated macrophage polarization and fibrotic remodeling in ectopic lesions

Endometriosis and adenomyosis are two distinct but related gynecological disorders marked by the presence of endometrial-like tissue outside its normal location. In endometriosis, this tissue grows outside the uterus, whereas in adenomyosis, it is found within the muscle layer of the uterus. Both conditions are associated with symptoms such as pelvic pain, abnormal bleeding, and fertility issues [218]. The etiology of endometriosis remains unknown, but it shares similar characteristics with tumors, such as abnormal glucose metabolism, indicating potential metabolic dysregulation [219]. Numerous glycolytic enzymes, such as 6-phosphofructo-2-kinase/fructose-2,6-bisphosphatase 3 (PFKFB3), are highly expressed in endometriosis cells, contributing to the progression of endometriosis [220]. Proviral insertion in murine lymphomas 2 (PIM2) promotes glucose consumption, lactate production, and the expression of glycolytic enzymes by increasing PKM2 expression, leading to fibrosis in endometriosis. Carboxyl terminus of Hsc70-interacting protein (CHIP) serves as a novel HMGB1 binding protein, targeting the ubiquitination and degradation of glycolysis-associated gene HMGB1, thereby inhibiting the proliferation and invasion of endometriosis [221]. Several glycolysis-related genes, such as HIF-1α, TGF-β, GLUT, PDK1, HK2, LDHA and PKM2, are highly expressed in endometriotic cells, promoting the development of endometriosis [222]. The differential expression of glycolysis-related genes in endometriosis tissue-derived cells leads to changes in glucose consumption and lactate production, which may be associated with inflammatory responses, hormonal changes, immune alterations, decreased oxygen tension, and elevated levels of free iron, all of which contribute to the pathogenesis of endometriosis [223].

Lactate, as a metabolic byproduct of glycolysis, increases cell migration, invasion, angiogenesis, and immune evasion during tumorigenesis, all of which are associated with the development of endometriosis. Therefore, the increased levels of lactate may contribute to the survival and establishment of endometriotic lesions, akin to the mechanism observed in cancer cell metastasis. Studies have confirmed a significant increase in lactate levels in peritoneal fluid from women with endometriosis and in stromal cells isolated from endometrial tissues. This elevated lactate production may provide energy for endometriotic cells, facilitating their survival, implantation, and invasion into the peritoneum, thereby contributing to the pathogenesis of endometriosis [223, 224]. Guo et al. discovered that lactate drives M2 polarization of macrophages in endometriotic lesions through the Mettl3/Trib1/ERK/STAT3 signaling pathway. This polarization results in immune-suppressive characteristics due to the abnormal secretion of chemokines and decreased phagocytic ability of macrophages [225]. Recent studies have explored the role of lactate-induced histone lactylation in the pathogenesis of endometriosis. Elevated levels of lactate and LDHA in endometriotic lesions enhance H3K18 lactylation in ectopic endometrial tissue and eutopic endometrial stromal cells (eESCs), promoting cell proliferation, migration, and in vitro invasion of endometriosis through upregulation of HMGB1 [226]. High expression of the lncRNA H19 in endometriosis promotes aerobic glycolysis and histone acetylation, thereby enhancing the proliferation and migration capacity of human endometrial stromal cells (HESCs) [227].

Metabolomic studies of follicular fluid in patients with endometriosis have shown elevated levels of pyruvate, lactate and glucose [228, 229]. This is interpreted as alterations in cellular glycolysis in follicular fluid associated with endometriosis due to the inflammatory processes during the disease. Enhanced anaerobic glycolysis in oocytes and granulosa cells is observed. As oocytes lack glucose transporters, they obtain energy from granulosa cells via lactate and pyruvate through gap junctions. Therefore, changes in the metabolic pattern of endometriotic granulosa cells directly impact oocyte development. Studies have shown mitochondrial dysfunction in granulosa cells of endometriosis patients, leading to decreased steroidogenesis, fertilization rates, oocyte maturation rates and oocyte quality [230]. To compensate for the energy metabolic defects caused by mitochondrial damage, granulosa cells tend to consume glucose through the glycolytic pathway, leading to increased glucose uptake and lactate accumulation. Unlike the pathogenic increase in glycolysis observed in endometriotic endometrial stromal cells, the increased glycolytic activity in granulosa cells can compensate for the energy shortage caused by mitochondrial dysfunction, thereby protecting granulosa cells from premature apoptosis. This adaptive change serves as a protective mechanism for female reproductive capacity [231].

Adenomyosis is a chronic gynecological condition with a wide-ranging prevalence, estimated between 8 to 62%, often coexisting with other pathologies such as uterine fibroids or endometriosis. It is typically characterized by abnormal uterine bleeding, dysmenorrhea, and infertility, negatively impacting the fertility, pregnancy outcomes, and quality of life of affected individuals [232]. Research has found that in women with adenomyosis, the expression of HK1, PFKFB3, GAPDH and PDHA mRNA in the ectopic uterine muscle layer is higher compared to the normal uterine muscle layer of women without adenomyosis. This indicates an increase in glycolysis and lactate accumulation in the ectopic uterine muscle layer of women with adenomyosis, which may lead to the infiltration of the endometrium into the uterine muscle layer and promote the development of adenomyosis [233].

### Polycystic ovary syndrome (PCOS): dysregulated glucose utilization and lactate signaling in follicular arrest and insulin resistance

Polycystic ovary syndrome (PCOS) is an endocrine and metabolic disorder that is marked by increased androgen levels and ovarian dysfunction. It affects approximately 8%−13% of women of reproductive age and is a leading cause of infertility. A hallmark of PCOS is insulin resistance (IR), which is frequently accompanied by compensatory hyperinsulinemia, affecting 50%−70% of women with the condition [234]. Insulin resistance refers to a diminished capacity of insulin to effectively regulate glucose uptake and production within the body. This condition can lead to downregulation of PI3K expression, thereby inhibiting the PI3K/AKT signaling pathway. As a result, the insulin-mediated regulation of glucose metabolism is impaired, disrupting the local glucose metabolism in the ovaries [235, 236]. Insulin controls the expression of GLUTs in granulosa cells, thereby regulating the uptake of glucose by oocytes and granulosa cells, leading to the accumulation of glucose in the local compartment (i.e., follicular fluid) [237]. In PCOS rats, there is a notable upregulation of GLUT1 expression in the ovaries, while the levels of key glycolytic enzymes, including HK and PFK, are significantly reduced. This suggests a diminished glucose utilization capacity in these animals. Furthermore, the expression of monocarboxylate transporters MCT2 and MCT4 is downregulated, leading to a decreased rate of transport for acetate and lactate [238]. Moreover, elevated androgens can inhibit the expression of LDHA, reducing lactate production. Crucially, elevated levels of nerve growth factor in the follicular fluid of PCOS patients lead to a significant reduction in the expression of LDHA. This impairment disrupts the communication between granulosa cells and oocytes, ultimately diminishing the developmental potential of the oocytes [239].

During follicular development, glycolytic activity increases, leading to higher lactate production as follicle diameter enlarges. However, studies have shown reduced lactate concentrations in the follicular fluid of PCOS patients, likely due to decreased glucose uptake and dysregulated LDH activity, which controls the conversion of pyruvate to lactate [240]. Follicular development in PCOS patients appears to require high lactate concentrations, and increasing lactate levels in follicular fluid in vitro may reduce the occurrence of follicular arrest [241, 242]. Research indicates that moderate insulin levels can promote lactate production in the follicular fluid of healthy individuals, but this effect is not significant in PCOS patients. High insulin doses, however, inhibit ovarian granulosa cells (KGN cells) and reduce lactate levels in these cells [243]. In PCOS, extracellular vesicle-derived microRNAs (miR-143-3p and miR-155-5p) silence glycolysis in KGN cells, lowering lactate production and weakening the environmental stimulus necessary for follicular development [244]. Recent studies have focused on targeting glycolysis and lactate metabolism abnormalities in PCOS to improve follicular development. Resveratrol (3,4,5-trihydroxy-trans-stilbene, RES) enhances ovarian insulin sensitivity, promotes granulosa cell glycolytic activity, and ameliorates the mechanism of follicular development impairment in PCOS rats. SIRT2 has been identified as a key regulator of RES-mediated glycolysis in ovarian granulosa cells [245]. Metformin treatment increases LDHA expression in the endometrial tissue of PCOS patients, reversing impaired uterine metabolism by inducing glycolysis and mitochondria-mediated cell death in the proliferative endometrium, which benefits both systemic and local uterine function [246]. The combination of Diane-35 (2 mg cyproterone acetate and 35 μg ethinylestradiol) with metformin improves glucose metabolism, reduces insulin resistance, lowers serum testosterone, and restores ovulation in PCOS patients. This therapeutic effect may be linked to the regulation of glycolysis-related mediators such as PKM2, LDHA, and SIRT1 [238].

### Embryo implantation: lactate as a critical bio-signaling hub for uterine receptivity and maternal–fetal immune tolerance

Implantation is a complex, multi-stage process involving embryo attachment, adhesion, and invasion into the appropriately prepared or "receptive" endometrium of the uterus. Proper embryo implantation into the maternal endometrium is crucial for establishing and developing a healthy placenta and ensuring a successful pregnancy. Studies indicated that approximately 30% of pregnancies end in miscarriage during the peri-implantation period in natural cycles; meaning a significant portion of naturally conceived human pregnancies fails to initiate, complete implantation, and achieve sustained pregnancy [247]. Before and after implantation, the blastocyst releases substantial amounts of lactate into the surrounding microenvironment. Blastocysts exhibit high levels of aerobic glycolysis, generating large quantities of lactate in the presence of oxygen. In addition to providing energy for blastocyst expansion and mitotic division, this high level of glucose utilization can synthesize triacylglycerols and phospholipids for new membrane synthesis, as well as serve as precursors for complex sugars such as glycoproteins and mucopolysaccharides. Furthermore, the oxygen concentration in the uterine cavity (1.5%−5.3%) is much lower than atmospheric levels (∼20%), and the oxygen availability during implantation, when the embryo invades the endometrium, is limited. Due to the absence of a maternal vascular system, the implantation site is relatively hypoxic. Under conditions of amino acid and carbohydrate metabolism, studies have found that lower oxygen concentrations are associated with upregulation of glucose metabolism and high levels of lactate formation [248]. In the late blastocyst and early implantation stages, the LDH isoform switches from LDHB (favoring pyruvate formation) to LDHA (favoring lactate formation) [249]. The high lactate microenvironment created by the blastocyst enables endometrial breakdown, angiogenesis, and immune modulation, facilitating successful implantation.

Implantation involves the degradation of the endometrial stroma, which facilitate the invasion of the underlying nutritive layer. Lactate production creates an acidic environment that enhances matrix metalloproteinase (MMP) activity and promotes the production of TGF-β. The resulting low pH reduces tissue inhibitors of metalloproteinases (TIMPs), increasing matrix degradation, and also activates tissue plasminogen activator (tPA) [250]. Lactate further stimulates hyaluronic acid synthesis, which aids in cell motility. These processes collectively support the invasion and implantation of the nutritive layer. After implantation, the establishment of maternal blood supply to the placenta requires angiogenesis, a process in which lactate plays a direct role. Elevated lactate levels prompt cellular uptake of lactate, activating growth factor pathways. Similarly, implanted embryos release lactate, which is absorbed by surrounding tissues. During implantation, the uterus produces high levels of VEGF in response to lactate stimulation, facilitating vasodilation, endothelial cell proliferation, migration, and ultimately, the formation of blood vessels [251]. Lactate from the blastocyst, through feedback loops, directly impacts uterine remodeling, immune regulation, and blastocyst function, including its survival via the NFκB pathway [252]. Preventing maternal rejection of the embryo is critical during implantation. Lactate modulates local immune responses during embryo growth and invasion, significantly reducing the proliferation and expression of T cell receptors and the production of cytokines by cytotoxic T cells. This suppression reduces the local immune response at the implantation site [253]. Additionally, lactate produced by the embryo can induce VEGF expression in macrophages, further promoting the implantation process [254].

Therefore, lactate serves as a crucial signaling molecule derived from the embryo, essential for successful implantation. Further research on the role of lactate in endometrial receptivity could improve our understanding of implantation failure, offering new opportunities to enhance the success rates of both natural and assisted pregnancy.

## Lactate metabolism serves as a diagnostic and prognostic indicator

Upstream and downstream factors of lactate metabolic pathways exhibit differential expression across various disease pathologies and actively contribute to disease initiation and progression. Concurrently, significant differences in lactylation modification sites in these diseases have been shown to have potential as markers for diagnosis and severity assessment [255, 256]. Increasing evidence suggests that targeting lactate metabolism-related regulatory factors and lactylation modification sites has gradually become a new strategy for clinical diagnosis and prognosis risk assessment. Tumor cells exhibit characteristic glycolytic activity, resulting in the secretion of large amounts of lactate into the extracellular microenvironment. The accumulation of lactate promotes the progression of malignant tumors, increases the risk of distant metastasis, and reduces patient survival rates. As lactate is the most significantly elevated metabolite in tumors, detecting lactate concentrations and related metabolites in tumor tissues or the microenvironment through relevant diagnostic methods aids in clinical diagnosis and prognosis assessment of the disease [257]. Zhu et al. identified nine lactate metabolism-related genes (PSMA2, TMEM258, BLOC1S1, LRP1, TXNDC17, HINT2, COA6, SOGA1 and DPM3) that significantly impact the prognosis of ovarian cancer. Based on these genes, they calculated a lactate score for patients, indicating that a high lactate score suggests poor prognosis [258]. Moreover, serum levels of LDH in ovarian cancer patients are notably elevated compared to those in individuals with benign ovarian tumors [259]. LDH levels show considerable variation across different stages and grades of ovarian cancer. Survival analyses reveal that elevated LDH expression correlates with reduced survival durations [260]. High expression of LDHA in cervical cancer and LDH5 in endometrial cancer is often indicative of poor overall survival and disease-free survival [261, 262]. LDH3 + (24/LDH1) is defined as the Uterine mass Magna Graecia (U.M.G.) Risk Index. An elevated U.M.G Risk Index (> 29) is associated with an increased likelihood of uterine sarcoma, demonstrating 100% sensitivity and 99.6% specificity for its diagnosis [263]. Furthermore, Niklasson et al. propose that the activity profile of LD isoenzymes in uterine fluid aspirates demonstrates 100% sensitivity and 85% specificity for the diagnosis of endometrial cancer in postmenopausal women. They assert that this method is more reliable than aspirate histology and can replace invasive procedures such as hysteroscopy or curettage with LD analysis [264]. The above studies indicate that LDH levels have the potential to serve as routine clinical pathological parameters for female reproductive system cancers.

As a predictor of mortality, lactate has been established as a reliable biomarker for prognosis in acute myocardial infarction (AMI), with elevated levels independently associated with adverse outcomes and mortality. Clinical data further demonstrate that lactate concentrations exceeding 1.8 mmol/L are significantly correlated with increased 30-day mortality in patients with ST-segment elevation myocardial infarction [98]. In terms of risk stratification, scoring systems incorporating lactate measurements, such as the CLIP score and IABP-SHOCK II risk score, markedly improve predictive accuracy, and dynamic changes in lactate levels (e.g., measurements at 8 h post-admission) provide even stronger prognostic information [265, 266]. Moreover, the application of lactate has extended to cardiac transplantation, where lactate levels in perfusion solutions correlate with graft quality and post-reperfusion function, offering a potential real-time assessment of donor heart viability [267]. In out-of-hospital cardiac arrest (OHCA), lactate levels are closely associated with patient survival and neurological recovery, providing critical metabolic guidance for clinical decision-making [268].

## Lactate and lactylation serve as a therapeutic target

Given the critical role of lactate in pathophysiological processes, lactate-targeted therapies have the potential to inhibit tumor growth and metastasis, as well as to treat inflammation-associated diseases. Currently developed clinical drugs targeting lactate metabolism primarily concentrate on (1) targeting glycolysis within cells, (2) targeting lactate transport, and (3) introducing exogenous substances to consume lactate. Additionally, lactylation modifications play a crucial role in metabolic reprogramming, immune regulation, and various physiological processes, underscoring its pivotal role in disease therapy and its potential as a critical target in epigenetics for disease intervention (Table 2).

**Table 2:** Therapeutic agents targeting lactate production, transport, and lactylation modifications

Targets	Drugs	Disease	Mechanism	Reference
LDHA	Oxamate	Cervical cancer	Lactate production	[271]
LDHA	JQ1	Ovarian cancer	Lactate production	[272]
LDHA	water-extracted *P. vulgaris*	Endometriosis	Lactate production	[276]
LDHA	GSK2837808A	Melanoma	Lactate production	[287]
LDHA	NHI-Glc-2	NSCLC and gastric cancer	Lactate production	[288]
LDHA	GNE-140	Pancreatic cancer	Lactate production	[289]
LDHA/B	MS6105	Pancreatic cancer	Lactate production	[290]
LDHA/B	NCI-006	Pancreatic cancer	Lactate production	[291]
MCT1/2	AR-C155858 (SR13801)	Breast cancers, B-cell lymphoma	Lactate production	[292]
MCT1	AZ3965	CRC, melanoma	Lactate excretion	[293]
MCT4	ALK-04	Melanoma	Lactate excretion	[294]
MCT4	α-CHCA	Ovarian cancer	Lactate excretion	[279]
MCT1, MCT4	Atorvastatin, resveratrol	endometriosis	Lactate excretion	[280]
Lactate	LOx	Breast cancer,	Lactate catabolism	[295]
Lactate	CoMnFe-LDO	UM	Lactate catabolism	[296]
HK2, PFKFB3	Meclizine	Adenomyosis	Glucose uptake	[297]
HK2, PDK1	Sodium selenite	Cervical cancer	Glucose uptake	[298]
LDHA, HK2, PKM2	Cryptotanshinone	Ovarian cancer	Lactate production, Glucose uptake	[188]
LDHA, HK2, PKM2	Ginsenoside F2	Cervical Cancer	Lactate production, Glucose uptake	[299]
LDHA, HK2, PKM2	*Dendrobium nobile-derived* polysaccharides	PCOS	Lactate production, Glucose uptake	[300]
Lactylation	K673-pe	Colorectal cancer	Non-histone modification	[301]
Lactylation	MG149	Colorectal cancer	Non-histone modification	[302]
Lactylation	D34-919	Glioblastoma	Non-histone modification	[84]
Lactylation	RJA	Hepatocellular carcinoma	Histone modification	[303]

LDHA catalyzes the formation of lactate from pyruvate, and inhibiting LDHA can suppress the Warburg effect, shifting the environment to one characterized by high glucose but low lactate levels, thereby controlling cancer progression. This strategy represents a primary target in disease therapy focused on lactate metabolism. Research indicates that LDHA inhibition can increase the sensitivity of drug-resistant cancers to other chemotherapy treatments [269]. Xiang et al. found that oxamate, a specific LDHA inhibitor, significantly enhances the inhibitory effect of PARP inhibitors on wild-type BRCA ovarian cancer [270]. In addition, Oxamate also significantly inhibits proliferation of cervical cancer cell lines [271]. JQ1, a selective small-molecule inhibitor of BET bromodomain proteins, has been found to reduce LDHA activity, inhibit lactate production, decrease energy supply to ovarian cancer cells, and inhibit tumor cell proliferation [272]. LDHA is considered a feasible target for drug design and discovery, with several small molecules showing significant LDHA inhibition and anticancer activity. However, to date, no reliable LDHA-specific inhibitors have been approved for clinical use to date. Mofetil (also known as AT-101), which targets and inhibits LDHA, is currently in preclinical trials for brain and central nervous system tumors, B-cell non-Hodgkin lymphoma, and other diseases, potentially becoming a future treatment option [273, 274].

In addition to LDHA, targeting other rate-limiting enzymes in glycolysis, such as PDH, HK2, 3PFKFB3 and PKM2, as well as glycolysis-related pathways, can effectively reduce lactate production in cells. Cryptotanshinone has been shown to decrease the expression of overactive glycolysis-related enzymes LDHA, HK2, and PKM2, and inhibit the STAT/SIRT3/HIF-1α signaling pathway, thereby suppressing glycolysis in ovarian cancer cells, inhibiting tumor cell growth, and inducing tumor cell apoptosis [188]. Targeting glycolysis has also been shown to combat drug-resistant diseases. The PFKFB3 inhibitor 3PO can be combined with cisplatin or paclitaxel to enhance the anti-proliferative effects of these chemotherapy drugs in ovarian cancer cells [275]. Water-extracted Prunella vulgaris significantly inhibits the expression of PDK1/3 and phosphorylation of PDHA, thereby suppressing aerobic glycolysis, inducing apoptosis, and reducing endometriosis lesions [276]. In patients with endometrial hyperplasia and PCOS, metformin treatment has been shown to normalize the abnormal expression of glycolytic enzymes and mitochondrial-associated proteins, leading to an improvement in endometrial receptivity [246]. Additionally, due to the downregulation of glycolytic pathway rate-limiting enzymes in PCOS patients, there is a decrease in ovarian glycolytic rates, requiring higher levels of pyruvate and lactate for normal follicular development. Administration of Resveratrol (3,4,5-trihydroxy-trans-stilbene, RES) and Mogroside V significantly upregulates the expression of these glycolytic rate-limiting enzymes. This action restores ovarian glycolytic activity, thereby improving disturbances in ovarian energy metabolism [245, 277]. Diane-35 (composed of 2 mg cyproterone acetate and 35 μg ethinyl estradiol) combined with metformin therapy is widely used in clinical treatment for PCOS. It has been demonstrated to upregulate glycolytic rate-limiting enzymes, thereby increasing ovarian lactate levels and improving follicular energy supply [238]. Furthermore, the glycolysis inhibitor 2-deoxy-D-glucose (2-DG), which competes with glucose, reduces lactate levels in vivo. Phase I clinical trials have demonstrated its clinical efficacy in various solid tumor patients, making it a promising candidate for clinical use [278].

MCTs are responsible for the transport of lactate in and out of cells. Therefore, MCT inhibitors can influence lactate metabolism and disease progression. For instance, blocking MCT4 with alpha-cyano-4-hydroxycinnamic acid (α-CHCA) disrupts lactate export in ovarian cancer cells, leading to decreased extracellular lactate levels. This shift reverses the epithelial-to-mesenchymal transition in ovarian cancer cells, inhibiting migration [279]. Atorvastatin and resveratrol significantly reduce MCT1 and MCT4 expression in ectopic endometrial tissues in rats, leading to decreased lesion size and reduced vascularization [280]. Studies have shown that knocking down MCT1 inhibits cancer cell proliferation and migration, thereby suppressing tumor progression [281]. Several MCT inhibitors with clinical potential are currently in preclinical trials, including AZD3965 (a pyrrolidine derivative targeting MCT1), fluvastatin (targeting MCT4), and diclofenac (targeting both MCT1 and MCT4).

Another therapeutic approach targeting lactate metabolism involves introducing exogenous substances to consume the produced lactate. Lactate oxidase (LOx) catalyzes the conversion of lactate to pyruvate without requiring a coenzyme, making it a naturally occurring enzyme with catalytic activity even higher than endogenous LDH. LOx, derived from various bacteria, holds significant therapeutic potential due to its irreversible lactate consumption. However, the challenge of achieving precise LOx delivery to lesions remains, as systemic administration can cause drug toxicity. To address this, various drug delivery systems, particularly nanomaterials, have been developed. Researchers have encapsulated LOx in nanocapsule, enabling precise and efficient delivery of LOx, enhancing its stability, and reducing systemic drug toxicity [282]. Additionally, some nanomaterials exhibit LDH-like activity, catalyzing lactate conversion, which holds significant research potential [283, 284].

The discovery of lactylation has further expanded the therapeutic strategies related to lactate metabolism. Given its critical role in disease pathogenesis, treatments targeting lactylation are promising and warrant further investigation. The p300/CBP proteins regulate lactylation levels, and inhibitors of p300, such as C646, CCS147, and EP3160, have been shown to reduce lactylation modifications of both histone and non-histone proteins. Although these inhibitors are still in clinical trial phases, they hold promise as potential therapeutic options. Additionally, various compounds have been demonstrated to lower lactylation levels. For instance, the triterpenoid anti-tumor compound demethylzeylasteral inhibits H3K9la and H3K56la lactylation [285]. Evodiamine has been shown to inhibit lactate-induced lactylation at the H3K18la site [286]. However, the specific targets of these compounds are still undetermined, and underlying mechanisms and additional compounds targeting lactylation sites warrant further exploration.

## Conclusion and future perspectives

In this review, we summarize the pathways and mechanisms of lactate production and clearance, as well as the biological functions of lactate metabolism. We further discuss the historical development of lactate research, the potential of lactate metabolism as a diagnostic and prognostic biomarker, and its promising prospects as a therapeutic target.

As a major product of glycolysis, lactate has increasingly been recognized for its multifaceted roles in both physiological and pathological processes, including energy metabolism, immune regulation, and signal transduction. The functions of lactate are inherently dualistic: it can promote immune evasion, yet some studies report beneficial effects on T cell function. Similarly, lactate can amplify inflammatory responses while also facilitating inflammation resolution. The paradoxical roles of lactate in disease warrant further investigation; however, for tumor- and inflammation-related disorders, therapeutic strategies primarily focus on promoting lactate clearance and inhibiting lactate production [198]. This duality complicates lactate-targeted therapies. In conditions such as PCOS, where ovarian lactate levels are reduced and energy metabolism is dysregulated, leading to impaired follicular development, supplementation of ovarian lactate represents a more appropriate intervention [245]. Nonetheless, challenges remain: would increasing lactate levels adversely affect tissues beyond the follicular microenvironment, and would lactate-lowering treatments for inflammatory diseases of the female reproductive system impact fertility in women of childbearing age? In cardiomyocytes, α-MHC-K1897la stabilizes sarcomeres and enhances contraction, whereas H3K18la primarily drives pathological hypertrophy, indicating that simple lactate supplementation or reduction may not provide an optimal solution. Accordingly, developing more precise and effective delivery strategies that selectively target specific lactate metabolic pathways and tissue sites is especially important. Nanomedicine-based targeted delivery of lactate metabolic modulators offers a safer and more reliable approach to minimize systemic toxicity. Current research has demonstrated promising progress in delivering modulators of MCTs, glycolytic rate-limiting enzymes, and LOX, highlighting their potential as therapeutic targets [282].

With the emerging focus on lactylation, our understanding of lactate's functions has reached new heights. Enzymes and genes associated with lactylation could become novel therapeutic targets beyond lactate metabolism pathways [39]. Nevertheless, several intriguing scientific questions remain to be addressed. First, although numerous studies have demonstrated dynamic changes in lactate levels across various disease processes, the temporal dynamics of these changes during disease progression remain poorly understood. Future research should employ multidimensional approaches, integrating spatial metabolomics, single-cell epigenomics, and functional validation, to delineate the precise roles of lactylation in disease pathogenesis. Second, the complete lactylation landscape remains unresolved in many diseases. Third, the specific mechanistic contributions of differentially expressed histone and non-histone lactylation to disease pathogenesis require further investigation. Beyond the established roles in enhancing protein expression, promoting nuclear gene accessibility, and stabilizing gene expression in other contexts, additional mechanisms may be involved. Finally, current research on lactylation primarily focuses on competitive inhibitors of p300/CBP, while specific lactylation “erasers” remain elusive. Therefore, the development of more targeted lactylation-modulating therapeutics represents a highly anticipated strategy for clinical intervention.

The critical roles of lactate metabolism-related genes in the progression of various diseases have also opened new avenues for disease diagnosis and prognostic assessment. Studies have shown that serum lactate dehydrogenase (LDH) levels are abnormally elevated in certain pathological conditions, highlighting the potential of lactate metabolism-related genes as non-invasive diagnostic biomarkers [304]. However, given lactate's involvement in numerous complex physiological processes, overly simplistic interpretations of lactate metabolism-related indicators could lead to significant misconceptions. A more nuanced understanding of lactate levels and their diverse functions, combined with the assessment of additional indicators, is required to improve the clinical relevance of these diagnostic and prognostic markers.

Lactate and its metabolic processes play a pivotal role in the initiation and progression of various diseases. Aberrations in lactate production, transport, and lactylation are key contributors to disease pathogenesis, while lactate levels can serve as valuable diagnostic and prognostic biomarkers. Strategies aimed at modulating lactate production and transport, regulating circulating lactate concentrations, and manipulating epigenetic modifications such as lactate-induced lactylation may offer innovative approaches for disease therapy.

## Data Availability

All relevant data are available from the authors upon request.

## Abbreviations

2-DG: 2-Deoxy-D-glucose
AD: Alzheimer's disease
AGR2: Anterior Gradient 2
AKT: Protein kinase B
ARG1: Arginase-1
ASCT2: Amino acid transporter type 2
ATP: Adenosine triphosphate
Aβ: Amyloid-β
BMDM: Bone marrow-derived macrophages
BRCA1: Breast cancer susceptibility gene 1
CAFs: Cancer-associated fibroblasts
cAMP: Cyclic AMP
CBP: CREB-binding protein
CHIP: Carboxyl terminus of Hsc70-interacting protein
COC: Cumulus-oocyte complex
DCA: Dichloroacetic acid
DNMT3A: DNA methyltransferase 3A
DOR: Diminished ovarian reserve
EC: Endometrial cancer
ECAR: Extracellular acidification rate
eESCs: Eutopic endometrial stromal cells
EMT: Epithelial-mesenchymal transition
EOC: Epithelial ovarian cancer
ESCs: Embryonic stem cells
ESRR-β: Estrogen receptor-related receptor beta
FBP1: Fructose-1,6-bisphosphatase 1
FBW7: F-box and WD repeat domain-containing 7
FSH: Follicle-stimulating hormone
G6PD: Glucose-6-phosphate dehydrogenase
G-CSF: Granulocyte colony-stimulating factor
GDH: Glutamate dehydrogenase
GLS1: Glutaminase 1
GLUTs: Glucose transporters
GM-CSF: Granulocyte-macrophage colony-stimulating factor
G-MDSCs: Granulocytic myeloid-derived suppressor cells
GPR81: G-protein coupled receptor 81
H3K18la: H3K18 lactylation
HCA1: Hydroxycarboxylic acid receptor 1
HDAC: Histone deacetylase
HESCs: Human endometrial stromal cells
HIF-1: Hypoxia-inducible factor 1
HIF-1α: Hypoxia-inducible factor 1-alpha
HK2: Hexokinase 2
HMGB1: High-mobility group box-1
IFN-α: Interferon-α
IFN-γ: Interferon-gamma
IGF2BP2: Insulin-like growth factor 2 mRNA-binding protein 2
iNOS: Inducible nitric oxide synthase
IR: Insulin resistance
ISGs: Interferon-stimulated genes
JAK1: Janus kinase 1
KIF23: Kinesin family member 23;
Kla: Lysine lactylation
LDH: Lactate dehydrogenase
LDHA: Lactate dehydrogenase A
LDHB: Lactate dehydrogenase B
LH: Luteinizing hormone
LMRGs: Lactate metabolism-related genes
Lox: Lactate oxidase
LPS: Lipopolysaccharide
MAVS: Mitochondrial antiviral signaling protein
MCTs: Glucose transporters
MCTs: Monocarboxylate transporters
MDSCs: Myeloid-derived suppressor cells
MDSCs: Myeloid-derived suppressor cells
NADH: Nicotinamide adenine dinucleotide
NSCLCs: Non-small-cell lung cancers
OC: Ovarian cancer
OCR: Oxygen consumption rate
OXPHOS: Oxidative phosphorylation
PCNA: Proliferating cell nuclear antigen
PCOS: Polycystic ovary syndrome
PDH: Pyruvate dehydrogenase
PDK1: Pyruvate dehydrogenase kinase 1
PD-L1: Programmed cell death ligand-1
PER1: Period circadian protein homolog 1
PFK: Phosphofructokinase
PFK1: Phosphofructokinase 1
PFKFB3: 6-Phosphofructo-2-Kinase/Fructose-2,6-Biphosphatase 3
PIM2: Proviral insertion in murine lymphomas 2
PITX2: Paired-like homeodomain transcription factor 2
PKM: Pyruvate kinase
POI: Premature ovarian insufficiency
PPP: Pentose phosphate pathway
PTM: Post-translational modification
RLR: RIG-I-like receptor
SN2: Sodium-coupled neutral amino acid transporter 5
SNHG9: Small nucleolar RNA host gene 9
TAMs: Tumor-associated macrophages
TAMs: Tumor-associated macrophages
Tas: Transaminases
TCA: Tricarboxylic acid
TIMM50: Translocase of inner mitochondrial membrane 50
TIMPs: Tissue inhibitors of metalloproteinases
TIMs: Tumor-infiltrating myeloid cells
TLR4: Toll-like receptor 4
TME: Tumor microenvironment
TP53: Tumor suppressor protein p53
Tpa: Tissue plasminogen activator
Tregs: Regulatory T cells
TRIM3: Tripartite motif-containing 3
UCEC: Uterine: corpus endometrial carcinoma
USP39: Ubiquitin-specific peptidase 39
VEGF: Vascular endothelial growth factor
XEN cells: Extraembryonic endoderm stem cells
α-CHCA: Alpha-cyano-4-hydroxycinnamic acid
α-KG: α-Ketoglutarate
